# Cardiomyocyte crosstalk with endothelium modulates cardiac structure, function, and ischemia-reperfusion injury susceptibility through erythropoietin

**DOI:** 10.3389/fphys.2024.1397049

**Published:** 2024-07-01

**Authors:** Jade P. Marrow, Razan Alshamali, Brittany A. Edgett, Melissa A. Allwood, Kyla L. S. Cochrane, Sara Al-Sabbag, Anmar Ayoub, Kjetil Ask, Gregory M. T. Hare, Keith R. Brunt, Jeremy A. Simpson

**Affiliations:** ^1^ Department of Human Health and Nutritional Sciences, University of Guelph, Guelph, ON, Canada; ^2^ IMPART Investigator Team Canada, Guelph, ON, Canada; ^3^ Faculty of Kinesiology, University of Calgary, Calgary, AB, Canada; ^4^ Firestone Institute for Respiratory Health, McMaster University, Hamilton, ON, Canada; ^5^ Department of Anesthesiology and Pain Medicine, St Michael’s Hospital, University of Toronto, Toronto, ON, Canada; ^6^ Department of Physiology, University of Toronto, Toronto, ON, Canada; ^7^ Keenan Research Centre for Biomedical Science in the Li Ka Shing Knowledge Institute, St. Michael’s Hospital, Toronto, ON, Canada; ^8^ Department of Pharmacology, Dalhousie Medicine New Brunswick, Saint John, NB, Canada

**Keywords:** erythropoietin, Cre-Lox, EPAS1 gene, hemodynamics, compensation, vascular endothelial growth factor, erythropoiesis

## Abstract

Erythropoietin (EPO) exerts non-canonical roles beyond erythropoiesis that are developmentally, structurally, and physiologically relevant for the heart as a paracrine factor. The role for paracrine EPO signalling and cellular crosstalk in the adult is uncertain. Here, we provided novel evidence showing cardiomyocyte restricted loss of function in *Epo* in adult mice induced hyper-compensatory increases in *Epo* expression by adjacent cardiac endothelial cells via HIF-2α independent mechanisms. These hearts showed concentric cellular hypertrophy, elevated contractility and relaxation, and greater resistance to ischemia-reperfusion injury. Voluntary exercise capacity compared to control hearts was improved independent of any changes to whole-body metabolism or blood O_2_ content or delivery (i.e., hematocrit). Our findings suggest cardiac EPO had a localized effect within the normoxic heart, which was regulated by cell-specific EPO-reciprocity between cardiomyocytes and endothelium. Within the heart, hyper-compensated endothelial *Epo* expression was accompanied by elevated *Vegfr1* and *Vegfb* RNA, that upon pharmacological pan-inhibition of VEGF-VEGFR signaling, resulted in a paradoxical upregulation in whole-heart *Epo*. Thus, we provide the first evidence that a novel EPO-EPOR/VEGF-VEGFR axis exists to carefully mediate cardiac homeostasis via cardiomyocyte-endothelial EPO crosstalk.

## 1 Introduction

Erythropoietin (EPO) is classically regarded as a renal-derived erythropoietic cytokine. In response to hypoxia, EPO stimulates the blast forming erythroid progenitor proliferation and survival to maintain hematocrit and/or restore oxygen delivery. Interestingly, non-renal sources of *Epo* are known to include the adult liver (Haidar et al., 1996), spleen (De Franciscis et al., 1979), ovaries (Masuda et al., 2000), uterus (Yasuda et al., 1998), testes (Magnanti et al., 2001), brain (Bernaudin et al., 1999; Weidemann et al., 2009; Urrutia et al., 2016), and heart (Wu et al., 1999; Kertesz et al., 2004; Chu et al., 2007; El Hasnaoui-Saadani et al., 2013). Within the heart, *Epo* production is generally ascribed to cardiomyocytes (Mengozzi et al., 2006; Miró-Murillo et al., 2011; El Hasnaoui-Saadani et al., 2013) and the endothelial lining (Mengozzi et al., 2006), yet the physiological significance of cardiac EPO is unclear. Since expression of the *Epo* receptor (*Epor*) occurs in multiple cardiac cell types (e.g., cardiomyocytes (Wright et al., 2004), endothelial cells (Beleslin-Cokic et al., 2004; Yang et al., 2014)), EPO likely elicits autocrine and/or paracrine cardiac-specific functional effects. While endothelial cells comprise ∼50% of the total number of cells in the murine heart, cardiomyocytes make up ∼30% (Pinto et al., 2016) and nearly 70% by total mass (Zak, 1973; Nag, 1980; Giordano et al., 2001). Thus, cardiomyocyte- and endothelial-derived factors as autocrine/paracrine effectors are vital for homeostasis. To date, the physiological role(s) of endogenously produced adult cardiomyocyte-derived EPO has not been investigated.

Recombinant human EPO (rhEPO) is neuroprotective (Bernaudin et al., 1999; Ponce et al., 2013; Bonnas et al., 2017; Wakhloo et al., 2020), cardioprotective (Cai et al., 2003; Calvillo et al., 2003; Parsa et al., 2003), and reportedly augments cardiac inotropy (Kaygisiz et al., 2006; Hefer et al., 2012) in prior pre-clinical studies. However, exogenous rhEPO is structurally distinct and differentially glycosylated compared to endogenous EPO. This can impact serum stability, receptor binding affinity, and bioactivity (Dube et al., 1988; Rahbek-Nielsen et al., 1997; Cheetham et al., 1998). Therefore, the beneficial effects of rhEPO cannot be inherently presumed to reflect endogenous, organ-specific functions of EPO. Instead, the pleiotropic roles of endogenous EPO should be assessed *in vivo* in a cell-specific context. Accordingly, we sought to investigate the impact of cardiomyocyte-restricted EPO signaling on cardiac autocrine/paracrine effects physiologically to contrast systemic or canonical erythropoietic functions determined using exogenous biology or receptor knock-out studies.

We previously generated a constitutive, cardiomyocyte specific *Epo* knockout mouse driven by the Mlc2v promoter (Allwood et al., 2024). When cardiomyocyte *Epo* is abolished during embryogenesis, cardiac cellular proliferation is reduced, leading to irreversible changes to overall morphology, function, and response to ischemic injury in the adult heart (Allwood et al., 2024). However, these mice also show a surprising transcriptional increase in endothelial cell derived *Epo* in compensation to loss of cardiomyocyte gene expression. This prohibits the distinction between developmental adaptations from physiological effects in the adult. To resolve whether cardiomyocyte-derived EPO signalling was physiologically relevant following a normal course of cardiogenic development, we used alpha-myosin heavy chain tamoxifen-induced Cre-LoxP loss of function in adult mice.

Herein we show that following the loss of cardiomyocyte derived *Epo* in the adult heart, compensatory hyperexpression persists by endothelial cells in a HIF2α-independent manner. This response was associated with concentric cellular hypertrophy, elevated contractile function, and a greater resistance to ischemia-reperfusion injury. Functionally, this phenotype translated to better voluntary exercise capacity, which was unrelated to changes in whole-body metabolism nor any change in hematocrit. Our findings suggest the overexpression of endogenous cardiac EPO acted locally, not systemically. In the absence of cardiomyocyte *Epo*, we observed concomitant upregulation of *Epo*, *Vegfr1*, and *Vegfb* RNA in the whole heart. When VEGF-VEGFR signaling was inhibited, a further increase in cardiac *Epo* could be observed. Collectively, our findings provide the first evidence for a paracrine cardioendothelial feedback loop by the EPO-EPOR/VEGF-VEGFR axis for maintaining cardiac homeostasis in the adult mouse.

## 2 Materials and methods

### 2.1 Ethical approval

Adult mice (C57Bl6 background) were bred and aged to 16 weeks of maturity for experiments and housed at 23°C–24°C with 45% humidity and maintained on a 12 h light/dark cycle with food and water provided *ad libitum*. This study was approved by the Animal Care Committee at the University of Guelph and all experiments were carried out in accordance with the guidelines from the Canadian Council on Animal Care.

### 2.2 Generation of EPO^Δ/Δ^ knockout mice

Inducible CreLox transgenic mice expressing *Epo* LoxP (Zeigler et al., 2010) and Cre recombinase under the control of the cardiomyocyte-specific promoter, alpha-myosin heavy chain (αMHC-MerCreMer), were used in this study (Sohal et al., 2001) (Jackson Laboratory Strain # 005657, Supplementary Figure S1). Briefly, the 5′loxP site was inserted into intron 1 of the *Epo* gene (located 94 base pairs upstream from exon), and the 3’ loxP site was inserted into intron 4 (located 86 base pairs downstream of the exon 4), with the NEO cassette flanked by both loxP sites.

Experimental mice were bred using the following schemes: female EPO LoxP without Cre (EPO^fl/fl^: αMHC-MerCreMer^−/−^) crossed with male EPO LoxP mice expressing homozygous Cre Recombinase (EPO^fl/fl^: αMHC-MerCreMer^+/+^), which resulted in 100% heterozygosity; female EPO^fl/fl^: αMHC-MerCreMer^+/+^ crossed with male EPO^fl/fl^: αMHC-MerCreMer^+/+^ (100% homozygous), female EPO^fl/fl^: αMHC-MerCreMer^+/−^ crossed with male EPO^fl/fl^: αMHC-MerCreMer^+/−^ (expected Mendelian genetics were 25% homozygous, 50% heterozygous, 25% wildtype) and female EPO^fl/fl^: αMHC-MerCreMer^+/−^ crossed with male EPO^fl/fl^: αMHC-MerCreMer^+/+^ (expected Mendelian genetics were 50% homozygous, 50% heterozygous). Genotyping used tail biopsies and PCR (REDExtract-N-Amp Tissue PCR Kit; Sigma-Aldrich, Oakville, ON, Canada) according to the manufacturer’s instructions. To confirm the presence of the floxed EPO alleles, the following primers sets were used: EPO-KO-F: 5′-AGT​GAA​GTT​TGG​CCG​AGA​AG-3’ (PCR Reaction A), EPO-KO-R: 5′- AGA​TCG​AAC​TTG​GCT​CCT​CA-3’ (PCR Reaction A), EPO-TAR-R: 5′-GTG​GGA​CGT​TCT​GGA​AGA​AA-3’ (PCR Reaction A). PCR Reaction–Stage 1: 1 Cycle, 95°C for 2 min; Stage 2: 40 Cycles, 95°C for 45 s; Annealing: 59°C for 1 min; Extension: 72°C for 1 min; Stage 3 Additional Extension: 1 Cycle, 72°C for 5 min; Hold at 4°C. Gel Electrophoresis–load 18.5 uL sample/well and run gel for 45 min at 90V (lights OFF). Expected Results–Homozygous EPO flox sites = one band at 344 bp; Heterozygote EPO flox sites = band at 228 bp and 344 bp; Wildtype (i.e., no flox) = one band at 228 bp. Representative agarose gel (1.5%) characterizing EPO floxing is presented in Supplementary Figure S1B.

For expression of Cre recombinase under the αMHC-MerCreMer promoter, the following primer sets and conditions were used: MerCreMer Common (Fwd): 5′-TCT​ATT​GCA​CAC​AGC​AAT​CCA-3’ (PCR Reaction A and B), MerCreMer Reverse: 5′-CCA​GCA​TTG​TGA​GAA​CAA​GG-3’ (PCR Reaction A), Wild Type Reverse: 5′-CCA​ACT​CTT​GTG​AGA​GGA​GCA-3’ (PCR Reaction B)**.** PCR Protocol for Reaction A and B–Stage 1: 1 Cycle, 95°C for 2 min; Stage 2: 40 Cycles, 95°C for 30 s; Annealing: 60°C for 30 s; Extension: 72°C for 1 min; Stage 3 Additional Extension: 1 Cycle, 72°C for 5 min; Hold at 4°C. Gel Electrophoresis–load 16 uL sample/well and run gel for 35 min at 95 V (lights OFF). Expected Results–Transgenic allele (αMerCreMer) = ∼300 bp; Heterozygote allele = 295 bp and ∼300 bp; Wild type allele = 295 bp. Representative agarose gel (1.5%) characterizing αMerCreMer Cre recombinase is presented in Supplementary Figure S1C.

At 8 weeks of age, EPO^fl/fl^: αMHC-MerCreMer^+/−^ mice were injected intraperitoneally (i.p.) with corn oil/tamoxifen mixture (25 mg/kg) once daily for 5 days to activate Cre recombinase and induce cardiomyocyte-specific deletion of *Epo* (denoted EPO^Δ/Δ^). Experiments were performed at 8 weeks post-injection (16 weeks old). Age-matched control mice (EPO^fl/fl^: αMHC-MerCreMer^−/−^, EPO^fl/fl^: αMHC-MerCreMer^+/+^ and EPO^fl/fl^: αMHC-MerCreMer^+/−^ without tamoxifen) demonstrated no differences and were subsequently combined (denoted EPO^fl/fl^). Cre-null mice provided tamoxifen (EPO^fl/fl^: αMHC-MerCreMer^−/−^ with tamoxifen) and mice without EPO floxed but with Cre (EPO^+/+^: αMHC-MerCreMer^+/−^ with and without tamoxifen) showed no major phenotypical differences compared to wildtype mice (Supplementary Table S1).

### 2.3 Voluntary wheel running

EPO^fl/fl^ and EPO^Δ/Δ^ mice were subjected to a 3-day voluntary wheel running protocol. Mice were housed individually and allowed to run freely on an in-cage wheel (12 cm in diameter). Rotations were transmitted to a cycling computer (VDO M2.1 WR Cycling Computer) and distance, time, and pace were recorded. Data were presented as the averages from all 3 days of voluntary wheel running following a 24-h acclimation period.

### 2.4 CLAMS: metabolic analyses

The Comprehensive Laboratory Animal Monitoring System (CLAMS) metabolic caging apparatus (Columbus Instruments Oxymax) is a sealed indirect calorimeter used for the simultaneous measurement of multiple parameters, including oxygen consumption (VO_2_), carbon dioxide production (VCO_2_), and calculation of respiratory exchange ratio (RER) across 24-h. EPO^fl/fl^ and EPO^Δ/Δ^ mice were weighed and individually placed into the CLAMS caging for a 24-h acclimatization period followed by a subsequent 24-h data collection period. Mice were maintained on a 12-h light/dark cycle and provided food and water *ad libitum*. Data was recorded every 15 min for metabolic readings (VO_2_, VCO_2_) and total energy expenditure. The RER was calculated as the quotient of VCO_2_/VO_2_.

### 2.5 Hematocrit and hemoglobin

For the determination of hematocrit, blood was collected from the left ventricle and saphenous vein of EPO^fl/fl^ and EPO^Δ/Δ^ mice and centrifuged in heparinized microcapillary tubes (5000rpm at 23°C for 10 min). To calculate hematocrit, the length of red blood cells was divided by the length of total blood volume and expressed as a percentage. To measure hemoglobin (g/L), saphenous vein blood was collected by microcuvettes and measured by a HemoCue^®^ Hb 201 (cat# 111716 Life Supply).

### 2.6 Echocardiography

Mice were anesthetized using an isoflurane/oxygen mix (2%/100%). Echocardiography was performed using the Vevo2100 system (VisualSonics Inc., Toronto, ON, Canada) with the 40 MHz MS550D ultrasound transducer. Mice were maintained at 37.5°C throughout data collection, as confirmed with a TH-5 rectal probe thermometer (Physiotemp Instruments LLC, Clifton, NJ, United States). B-Mode images were captured from the parasternal long axis mid-papillary region to measure the left ventricle endocardial length (i.e., LV chamber length) from the aortic annulus to the apex during in diastole, as previously described (Vinhas et al., 2013). M-Mode images were collected from the parasternal long axis mid-papillary region and analyzed using the left ventricle trace function from the cardiac package (VisualSonics Inc., Toronto, ON, Canada) as previously described (Platt et al., 2017). Measurements represent the average data collected over three consecutive cardiac cycles. Data collection and analyses were performed in a blinded manner.

### 2.7 Invasive hemodynamics

Cardiac function was investigated *in vivo* by invasive hemodynamics. Mice were anaesthetized using an isoflurane/oxygen mix (2%:100%). Animals were maintained at 37.5°C throughout data collection using a heated water pad. A 1.2F catheter (FTS-1211B-0018; Transonic Scisense Inc.) was inserted into the right carotid artery and advanced into the left ventricle to collect hemodynamics measurements. Hemodynamics were collected for 15 min. All hemodynamic signals were digitized at a sampling rate of 2000 Hz and recorded by computer using iWorx^®^ analytic software (Labscribe2, Dover, NH, USA). Data collection and analyses were performed in a blinded manner. Tissues were collected, weighed, and randomly assigned to either histology, qPCR, or western blotting.

### 2.8 Langendorff preparation

The *ex vivo* Langendorff preparation provides the direct assessment of systolic and diastolic cardiac function (and its susceptibility to ischemia-reperfusion injury), independent of preload, afterload, and heart rate, without the influence of neurohormonal effects or humoral factors in the blood. Accounting for these variables allows for conclusions to be made about purely intrinsic cardiac function. Mice were heparinised (200 IU/kg of body weight) for *ex vivo* cardiac assessment. After 20 minutes, mice were anesthetized using isoflurane, followed by a midline thoracic incision made to rapidly excise the heart. The heart was rinsed in ice-cold phosphate buffered saline and the aorta was cannulated onto a 21-gauge needle to allow for retrograde perfusion. Hearts were perfused with carbogenated (95% O2: 5% CO2) Krebs Henseleit buffer (pH of 7.4) at 70–75 mmHg. The Krebs Henseleit buffer contained the following compounds (in mM/L): 118 mM NaCl, 4.7 mM KCl, 1.2 mM MgSO4, 1.2 mM KH2PO4, 0.5 mM C3H3NaO3, 0.05 mM EDTA, 11 mM glucose, and 2 mM CaCl2. The left atrium was removed to allow the insertion of a deflated balloon attached to a pressure catheter into the left ventricle. The balloon was inflated to achieve an end diastolic pressure of 5–8 mmHg. Hearts were paced using a Grass SD9 Stimulator at a frequency of 7 Hz. A stabilization period of 20 min was followed by recording of baseline measurements, followed by 25 min of global no-flow ischemia. Afterwards, the perfusate line was reopened and hearts were re-perfused for 45 min. To assess cytoprotective function *ex vivo*, percent recovery of left ventricular pressure, and rate of change in pressure (dP/dt_max_–index of contractility/inotropy, and dP/dt_min_–index of relaxation) were calculated after 45 min of reperfusion. Data collection and analyses were performed in a blinded manner.

### 2.9 Serum EPO ELISA

Blood was collected from the left ventricle via cardiac puncture and allowed to clot for 2 h on ice, then centrifuged in 1.5 mL Eppendorf tubes at (4,000 rpm at 4°C for 20 min). The supernatant was removed, snap frozen in liquid nitrogen, and stored at −80°C for quantification of serum EPO levels. EPO protein concentration was quantified from serum using a Quantikine Mouse EPO ELISA (MEP00B, R&D Systems) according to the manufacturer’s instructions.

### 2.10 qPCR molecular analyses

Tissues selected for qPCR were snap frozen in liquid nitrogen and kept at −80°C (n = 8 per group for EPO^fl/fl^ and EPO^Δ/Δ^, n = 7 EPO^fl/-^ Cre^+/−^). Total RNA was isolated from the free left ventricle and kidneys using TRIzol (Invitrogen, Burlington, ON, Canada) with the Qiagen RNeasy kit (Qiagen, Hilden, Germany) according to the manufacturer’s instructions. RNA samples were treated with DNase (Qiagen), according to manufacturer’s instructions. Prior to cDNA synthesis, RNA concentrations were quantified (NanoDrop, ND1000; Thermo Fisher Scientific, Waltham, Massachusetts, USA). Protein contamination was assessed by measuring absorbance at 280 nm. Generation of cDNA was completed using a High-Capacity cDNA Reverse Transcription Kit (Applied Biosystems by Thermo Fisher Scientific, Waltham, Massachusetts, USA) according to the manufacturer’s instructions, using 2000 ng of RNA per sample. Target gene RNA was quantified using the Platinum SYBR Green qPCR SuperMix-UDG with ROX (Invitrogen, Burlington, ON, Canada) using the primers listed in Table 1. Primers were designed to span an exon-exon region to eliminate any possibility of priming genomic contamination. qPCR was performed using 7,500 Real Time PCR detection system (Applied Biosystems, Foster City, CA, USA) with the following protocol: 1 cycle at 50°C for 2 min, 1 cycle at 95°C for 5 min, then 40 cycles at 95°C for 15 s, 1 min at 60°C for all genes (excluding *Epo*, where the annealing temperature was 58°C), followed by a dissociation curve to assess specificity of the reaction (Supplementary Figure S2). Samples were run in duplicate 25 uL reactions. Undetectable *Epo* RNA was assigned the value of the limit of detection of the assay (CT = 40). Results were analyzed according to the delta-delta CT method using reference genes (Table 1) and normalized to the EPO^fl/fl^ group.

**TABLE 1:** List of mouse qPCR primers and sequences. *Epo*, erythropoietin; *Vegf*, vascular endothelial growth factor; *Hif*, hypoxia inducible factor; *Hmox-1*, heme oxygenase 1; *Pgk-1*, phosphoglycerate kinase 1; *Glut-1*, glucose transporter 1; *Cas8*, caspase 8; *αMhc*, alpha myosin heavy chain; *βMhc*, beta myosin heavy chain; *Anp*, atrial natriuretic peptide, *Bnp*, brain natriuretic peptide; *Eef1e1*, Eukaryotic Translation Elongation Factor 1 Epsilon 1; *Rpl32*, ribosomal protein L32; *β-Actin*, beta-actin.

Target	Primer Name	Sequence (5′-3′)	Annealing temperature (°C)
*Epo*	EPO-F	CAT​CTG​CGA​CAG​TCG​AGT​TCT​G	58
	EPO-R	CAC​AAC​CCA​TCG​TGA​CAT​TTT​C	58
*Vegfa*	VEGF-A-F	GGA​GAC​TCT​TCG​AGG​AGC​ACT​T	60
	VEGF-A-R	GGC​GAT​TTA​GCA​GCA​GAT​ATA​AGA​A	60
*Vegfb*	VEGF-B-F	TCT​GAG​CAT​GGA​ACT​CAT​GG	60
	VEGF-B-R	TCT​GCA​TTC​ACA​TTG​GCT​GT	60
*Vegfr1*	VEGFR-1-F	TGG​ACC​CAG​ATG​AAG​TTC​CC	60
	VEGFR-1-R	GCG​ATT​TGC​CTA​GTT​TCA​GTC​T	60
*Vegfr2*	VEGFR-2-F	GAG​AGC​AAG​GCG​CTG​CTA​GC	60
	VEGFR-2-R	GAC​AGA​GGC​GAT​GAA​TGG​TG	60
*Vegfr3*	VEGFR-3-F	CTG​GCA​AAT​GGT​TAC​TCC​ATG​A	60
	VEGFR-3-R	ACA​ACC​CGT​GTG​TCT​TCA​CTG	60
*Hif1α*	HIF-1α-F	CCC​ATT​CCT​CAT​CCG​TCA​AAT​A	60
	HIF-1α-R	TTACGCATGGCCGTTTCT	60
*Hif2α*	HIF-2α-F	CAG​CTT​CCT​TCG​GAC​ACA​TAA	60
	HIF-2α-R	CTC​CAA​GGC​TTT​CAG​GTA​CAA	60
*Hmox-1*	HO-1-F	GGT​GAT​GGC​TTC​CTT​GTA​CC	60
	HO-1-R	AGT​GAG​GCC​CAT​ACC​AGA​AG	60
*Pgk-1*	PGK-1-F	CAC​AGA​AGG​CTG​GTG​GAT​TT	60
	PGK-1-R	CTT​TAG​CGC​CTC​CCA​AGA​TAG	60
*Glut-1*	GLUT-1-F	GGT​GTG​CAG​CAG​CCT​GTG​TAC​G	60
	GLUT-1-R	TAG​GAC​ATC​CAA​GGC​AGC​CGT​TC	60
*Cas-8*	Cas-8-F	TGC​TTG​GAC​TAC​ATC​CCA​CAC	60
	Cas-8-R	TGC​AGT​CTA​GGA​AGT​TGA​CCA	60
*αMhc*	αMHC-F	CAC​CAA​CAA​CCC​ATA​CGA​CTA​C	60
	αMHC-R	TCA​GCA​CAT​CAA​AGG​CAC​TAT​C	60
*βMhc*	βMHC-F	AGA​TGG​CTG​GTT​TGG​ATG​AG	60
	βMHC-R	TTG​GCC​TTG​GTC​AGA​GTA​TTG	60
*Anp*	ANP-F	GGG​TAG​GAT​TGA​CAG​GAT​TGG	60
	ANP-R	TTC​CTC​CTT​GGC​TGT​TAT​CTT​C	60
*Bnp*	BNP-F	GGG​AGA​ACA​CGG​CAT​CAT​T	60
	BNP-R	CCC​AGC​GGT​GAC​AGA​TAA​AG	60
*Eef1e1*	Eef1e1-F	TAA​CAT​CAC​CCT​GGC​GGA​CA	60
	Eef1e1-R	TGA​CAA​AAC​CAG​CGA​GAC​ACA	60
*Rpl32*	Rpl32-F	GCC​TCT​GGT​GAA​GCC​CAA​G	60
	Rpl32-R	TTG​TTG​CTC​CCA​TAA​CCG​ATG​T	60
*β-Actin*	β-Actin-F	TGT​GAT​GGT​GGG​AAT​GGG​TCA​GAA	60
	β-Actin-R	TGT​GGT​GCC​AGA​TCT​TCT​CCA​TGT	60

### 2.11 RNA fluorescent *in situ* hybridization

The hearts were excised and block fixed in 10% buffered formalin for 24 h, followed by transfer into 70% ethanol for storage before processing and embedding in paraffin wax. Tissue microarrays were created using the tissue micro array (TMA) Master II instrument from 3D Histech Ltd. Regions of interest from the left ventricle were selected, cored, and added into a host paraffin block to create an array of 84 tissue cores, each measuring 2.0 mm in diameter (biological replicates n = 3).

To study *Epo* gene mRNA expression and localization from cardiac cell types, the microarray sections were stained using the following fluorophores: *Epo* with FITC (channel 2, accession # NM_007942.2) using RNAScope 2.5 LS Probe Mm-Epo-C2 Cat. No. 315508-C2, and with Cy5 (channel 1, accession # NM_007942.2) using RNAScope 2.5 LS Probe Mm-Epo-01 Cat. No. 444948. Then, kinase insert domain receptor (*Kdr*), an endothelial cell marker, was stained with Cy5 (channel 1, accession # NM_01612.2) using RNAScope 2.5 LS Probe-Mm-Kdr Cat. No. 414818. Myosin heavy chain 6 (*Myh6*), a cardiomyocyte marker, was stained with TRITC (channel 3, accession # NM_001164171.1) using RNAScope 2.5 LS Probe-Mm-Myh6 Cat. No. 506258. Finally, 4′,6-diamidino-2-phenylindole (*Dapi*) was used to stain nuclei. *In-situ* hybridization duplex staining was performed using commercially available assays and the ACD Bio program for the Leica Bond RX immunostainer. The slides were scanned using an Olympus VS 120 automated slide scanner, and images of 1 mm x 1 mm were acquired at ×40 magnification. The acquired images were analyzed using the HALO image analysis software from Indica Labs (v3.2.1851.229).

### 2.12 Histology

Mice were exsanguinated and 10 mL of 1x PBS, 10 mL of 0.5 mol/L KCl, and 10 mL of 10% buffered formalin (VWR, Mississauga, ON, Canada) were perfused through the right carotid artery to fix cardiomyocytes in diastole. Tissues were harvested, stored in 10% buffered formalin for 24 h, and then transferred into 70% ethanol. Hearts were processed and embedded in paraffin wax. Cross sections of the heart (5 μm) were mounted onto charged 1.2 mm Superfrost slides (Fisher Scientific). Paraffin embedded sections were then stained with either hematoxylin and eosin or Picrosirius Red for the determination of cardiomyocyte cross-sectional area (CSA) or percent fibrosis, respectively, within the left ventricle. Bright field images were acquired using an Olympus FSX 100 light microscope and analyzed blinded using ImageJ (for CSA) and cellSens (for interstitial fibrosis).

### 2.13 CoCl_2_ injections

Cobalt chloride (CoCl_2_) is used as a hypoxia mimetic for its ability to chemically induce HIF-α stabilization and upregulate downstream HIF target genes under normoxic conditions (reviewed and discussed in detail (Muñoz-Sánchez and Chánez-Cárdenas, 2019; Packer, 2020)). The effects of CoCl_2_ are widespread, resulting in HIF-2α stabilization across multiple cell populations and tissues, including the myocardium (Wiesener et al., 2003). Mice were injected i. p. with either CoCl_2_ (30 mg/kg) dissolved in 1 mL sterile PBS (to simulate hypoxia) or vehicle control (PBS). Hearts were collected for HIF-2α western blotting at the following endpoints: 45 min, 1.5 h, or 3 h after injection.

### 2.14 Immunoblotting

For probing of HIF-2α, left ventricular samples were homogenized in a buffer with a phosphatase (PhosSTOP, cat. # 4906845001, Sigma) and protease inhibitor cocktail (cat. #P8340, Sigma), and separation of nuclear from cytoplasmic extracts was performed using the NE-PER kit as per the manufacturer’s instructions (cat. # 78833, Life Technologies). Nuclear protein extract concentrations were measured by bicinchoninic acid assay (cat. # 23277, Fisher Scientific). Samples were equally loaded (20ug/well) and separated by 10% SDS-PAGE, followed by immunoblotting. Nitrocellulose membranes were rinsed in ddH2O and then incubated in reversible Ponceau Stain for 7 min to confirm equal protein transfer. The stain was stripped using 200uM NaOH for 1 min and rinsed in ddH2O for 5 min x 3. Membranes were blocked (5% skim milk in 1x TBST (0.1% tween)) and incubated in a primary HIF-2α antibody (Sun et al., 2015) (1:1000; cat. # NB100-122SS, Bio-Techne; 5% bovine serum albumin in 1x TBST) overnight at 4°C. Membranes were washed for 5 min x 3 in TBST and then incubated with a goat anti-rabbit IgG horseradish peroxidase–conjugated secondary antibody (1:1000, cat. # HAF008, Bio-Techne; 1% skim milk for 1 h at 22°C). Membranes were washed for 5 min x 3 in 1x TBST. Signal was detected and quantified via enhanced chemiluminescence (cat. # 1705060, Bio-Rad) using a FluorChem HD imaging system (Alpha Innotech, Santa Clara, CA, USA). Values were obtained by measuring the target band (normalized to Ponceau) relative to the EPO^fl/fl^ group.

### 2.15 Treatment with VEGF receptor tyrosine kinase inhibitor, axitinib

Axitinib is an FDA-approved selective inhibitor of cellular phosphorylation of VEGF receptor tyrosine kinases (VEGFR-1, VEGFR-2, VEGFR-3) for the treatment of advanced renal cell carcinoma (Tyler and FCSHP, 2012; INLYTA, 2023). Axitinib (cat. #S1005, Selleck Chemicals) was prepared as previously described (Ma and Waxman, 2009). Briefly, axitinib was suspended at 5 mg/mL in polyethylene glycol 400 (cat. # PX1286B-2, Sigma) and sonicated at room temperature for 30 min until dissolved. Using 0.1N HCl, the pH was adjusted to 2.5, followed by a second round of 10-min sonication. To achieve a final ratio of 3:7 (v/v) of polyethylene glycol 400 to water, acidified water (pH 2.5) was added. The solution was prepared fresh and stored in the dark at 4°C. Axitinib was administered once a day through i. p. injection at a dose of 25 mg/kg body weight in a volume of 5 μL/g body weight (Ma and Waxman, 2009) for 4 days x 2 cycles (with 2 days rest). Data (invasive hemodynamics, saphenous vein hemoglobin levels, and tissues for qPCR) were collected 24 h after the last injection.

### 2.16 Cell apoptotic assay

A one-step TUNEL *in situ* apoptosis kit (cat. # E-CK-A320, Elabscience Biotechnology Co.) was used to quantify differences in cell apoptosis between EPO^fl/fl^ and EPO^Δ/Δ^ hearts according to the manufacturer’s instructions. Fluorescent images were acquired using an Olympus FSX 100 microscope and the amount of green fluorescence of TUNEL-positive cells was analyzed blinded using ImageJ.

### 2.17 Statistical analyses

Graphing and statistical analyses of the data presented was performed using Prism version 9 software developed by (GraphPad Inc., La Jolla, CA, USA). Power calculations were used to determine the number of mice needed to detect a significant effect. Results were reported as mean ± SD (morphometrics, serum, hematocrit, qPCR, western blotting) or mean ± SEM (CLAMS, echocardiography, histology, invasive hemodynamics, Langendorff). To confirm whether data was normally distributed, a Shapiro-Wilk test was used. If the data was normally distributed, either a one-way ANOVA followed by Dunnett’s or Tukey’s post-hoc test or an unpaired Student’s t-test was performed. If the data was not normally distributed, a Kruskal–Wallis test with Dunn’s post-hoc test or Mann-Whitney *U* test was performed. Simple linear regression correlation analyses were run comparing *Epo* RNA expression to dP/dt_max_, dP/dt_min_, and dP/dt@LVP40 values and goodness-of-fit values were provided. *p*-value <0.05 was considered significant.

## 3 Results

### 3.1 Cardiomyocyte-specific deletion of *Epo* induced compensatory overexpression of *Epo* in the heart by endothelial cells

**FIGURE 1:**
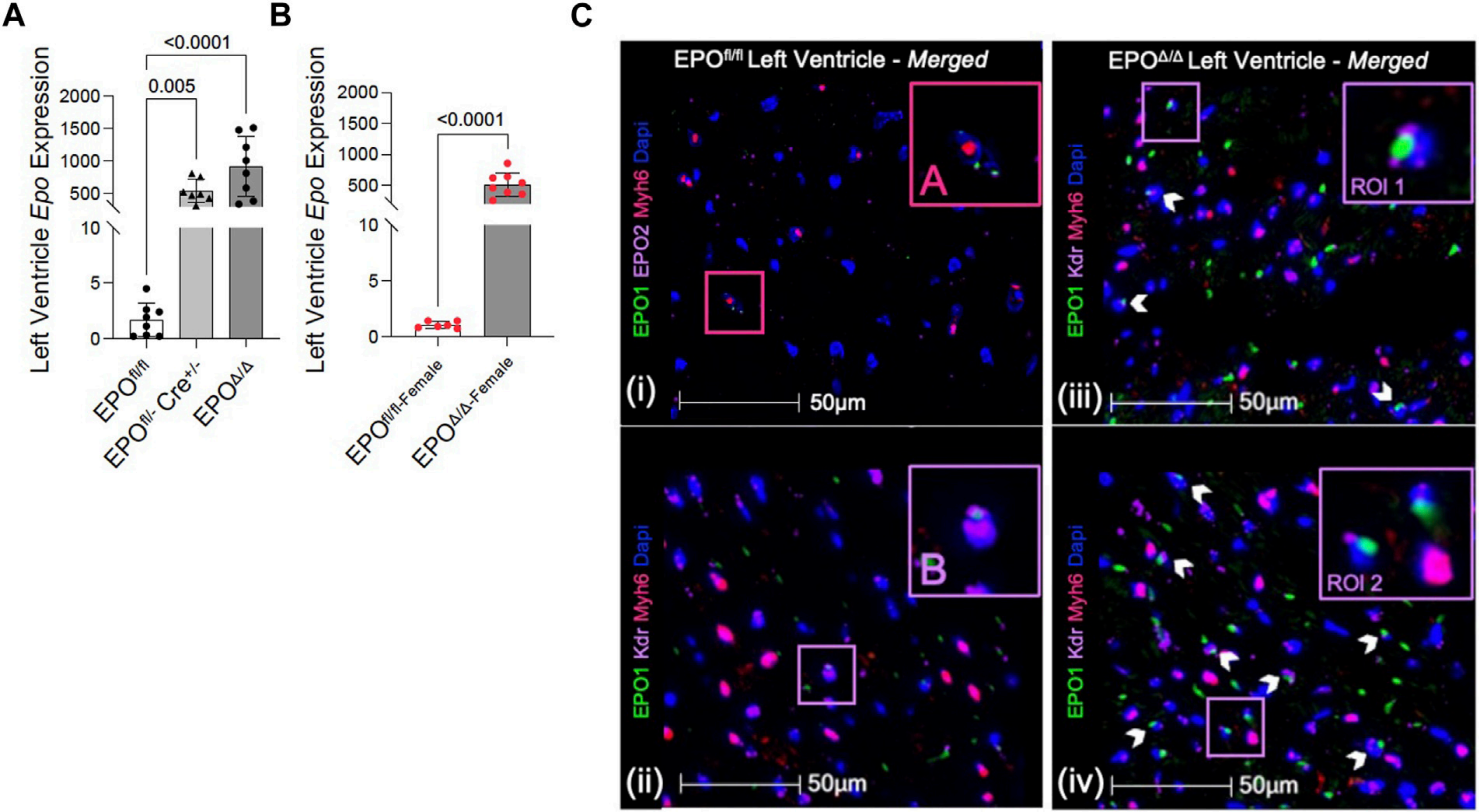
*Epo* RNA expression was significantly upregulated in male and female EPO^Δ/Δ^ mice by qPCR. **(A)** Left ventricular expression of *Epo* RNA (normalized to *Eef1e1*) of EPO^fl/fl^, EPO^fl/- Cre+/−+TAM^, and EPO^Δ/Δ^ mice. **(B)** Left ventricular expression of *Epo* RNA (normalized to *Rpl32*) of female EPO^fl/fl^ and EPO^Δ/Δ^ mice. Panels **(Ci-iv)**: Cardiomyocytes and endothelial cells contributed to basal *Epo* expression in EPO^fl/fl^, which upon cardiomyocyte specific *Epo* deletion (EPO^Δ/Δ^), became overcompensated by the endothelial cells, leading to hyper-expression. **(i)** A cardiomyocyte (*Myh6*, TRITC) and **(ii)** endothelial cell (*Kdr*, Cy5) show co-localization with EPO (FITC, green) in EPO^fl/fl^. However, in EPO^Δ/Δ^ mice, the cardiomyocyte does not appear to be a contributing source, confirming successful knockout of EPO. Rather, **(iii, iv)** show two regions of interests (RIO) to highlight intense endothelial-derived EPO signal. The pink box “**(A)**” is used to show cardiomyocyte-*Epo* colocalization in EPO^fl/fl^, purple box “**(B)**” is used to show endothelial-*Epo* colocalization in EPO^fl/fl^, purple boxes “ROI 1” and “ROI 2” show upregulated EPO signal from endothelial cells. A nuclei marker was used (*Dapi*, blue). Scale bar represents 50 µm. A one-way ANOVA followed by a Dunnett’s post-hoc test was used to detect differences in left ventricular expression between EPO^fl/fl^, EPO^fl/- Cre+/−+TAM^, and EPO^Δ/Δ^ mice. An unpaired, two-tailed t-test was used to detect a difference between EPO^fl/fl-female^ and EPO^Δ/Δ-female^. Data are expressed as mean ± SD and were considered significant when *p* < 0.05.

**FIGURE 2:**
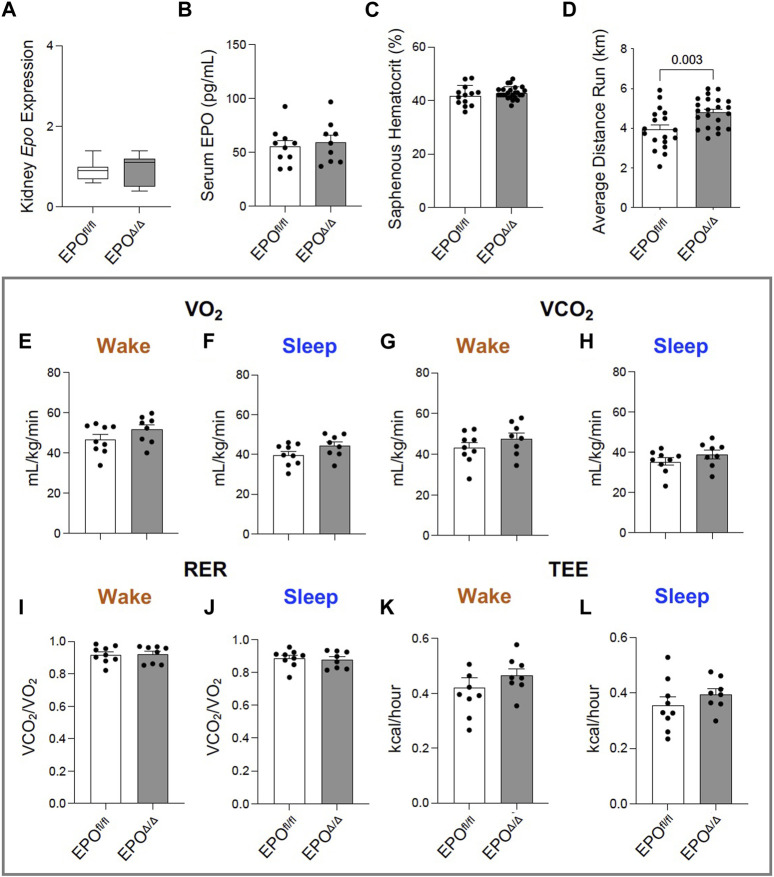
Greater voluntary wheel running capacity in EPO^Δ/Δ^ compared to EPO^fl/fl^ mice occurred independent from differences in hematocrit and whole-body metabolism. **(A)** Kidney *Epo* RNA expression (normalized to *β-Actin*) in EPO^fl/fl^ and EPO^Δ/Δ^ mice, **(B)** serum EPO, **(C)** hematocrit (%) collected from the saphenous vein of EPO^fl/fl^ and EPO^Δ/Δ^ mice, and **(D)** average voluntary wheel running distance (km) across 3 days. Comprehensive Laboratory Animal Monitoring System (CLAMS) measured **(E, F)** VO_2_ (mL/kg/min), **(G, H)** VCO_2_ (mL/kg/min), **(I, J)** RER (VCO_2_/VO_2_), and **(K-L)** total energy expenditure (TEE, kcal/hour) in EPO^fl/fl^ and EPO^Δ/Δ^ mice during their sleep and wake phases. An unpaired, two-tailed t-test was used to detect differences. Data are expressed as mean ± SD (for qPCR, serum EPO levels, and hematocrit) or mean ± SEM (for average running distance and whole-body metabolic readings). Data were considered significant when *p* < 0.05.

In the developing fetus, mice null for *Epo* and the *Epor* die by E13.5 due to impaired cardiogenesis and anemia (Wu et al., 1999; Kertesz et al., 2004). Our previous work using the Mlc2v promoter established cardiomyocyte specific *Epo* deletion during embryogenesis in mice induces a phenotype with less cardiomyocyte hypoplasia, compensatory cellular hypertrophy, and upregulation of *Epo* in the heart by the endothelial cell persisting into adulthood (Allwood et al., 2024). Therefore, the presence of cardiac EPO is critical for proper development of the heart, yet endogenous cardiac *Epo* expression, regulation, and physiological significance in the adult heart remains unknown. For this reason, in our follow up study, we hypothesized that the demand for compensatory cardiac EPO would be reduced in a fully, and normally, developed adult heart. Upon induced deletion of adult cardiomyocyte *Epo* in mice after an ordinary course of cardiogenesis, we expected whole-heart morphology to be normal and cardiac *Epo* production to be low. To verify this, mice were subjected to morphometric and quantitative tissue PCR analysis. In line with our previous reports (Allwood et al., 2024), the morphological measurements revealed no gross differences in body weight, heart weight, left ventricular weight, or heart weight/tibial length (HW/TL) ratio between groups (Supplementary Figure S3C; Table 2). Surprisingly, after tamoxifen induced MerCreMer deletion of *Epo* expression, there was a significant upregulation in cardiac *Epo* in EPO^Δ/Δ^ mice compared to the EPO^fl/fl^ group by qPCR (Figure 1A). This data was confirmed by a mildly graded response in mice with only *one* floxed EPO allele (i.e., EPO^fl/-^ Cre^+/−^) (Figure 1A), suggesting this phenomenon does not exist in an all-or-nothing manner. Further, we sought to confirm our data was not specific to male mice. Indeed, female EPO^Δ/Δ^ mice also showed a significant increase in whole-heart *Epo* upon targeted cardiomyocyte *Epo* deletion (Figure 1B) with no apparent sex effect (Supplementary Figure S4). To identify the specific cell type(s) responsible for *Epo* expression under normoxic conditions in EPO^fl/fl^ and EPO^Δ/Δ^ mice, we used RNA fluorescent *in situ* hybridization. As expected, we observed low basal *Epo* mRNA production by the cardiomyocyte (Figure 1Ci) and endothelial cell (Figure 1Cii) in EPO^fl/fl^ mice. By contrast, in the EPO^Δ/Δ^ mice, an over-abundance of *Epo* signal (shown using two regions of interest, Figure 1Ciii, iv) was co-localized with the endothelial cells, indicating that upon successful cardiomyocyte-*Epo* knockout, the endothelium compensated for the loss by increasing its own *Epo* expression. Therefore, not only have we verified this phenomenon in the adult mouse using a second independent CreLox line to support our earlier work (Allwood et al., 2024), but we also showed hyper-compensated cardiac *Epo* occurs regardless of floxed allele homozygosity (EPO^Δ/Δ^) or heterozygosity (EPO^fl/-^: Cre^+/−^), *and* in both sexes. Importantly, despite marked *Epo* overexpression in the adult EPO^Δ/Δ^ mouse, on a whole-body and organ level, there were no visual abnormalities (Supplementary Figure S3A). There was also no change in renal *Epo* expression (Figure 2A), serum EPO (Figure 2B), or hematocrit (Figure 2C), suggesting that there would be no altered physiological consequences from compensatory cardiac endothelial overexpression.

**TABLE 2:** Morphometrics. HW, heart weight; TL, tibial length; LV, left ventricle peak pressure. Data is presented as mean ± SD.

	EPO^fl/fl^ (n = 35)	EPO^Δ/Δ^ (n = 39)	*p*-value
**Body Weight (g)**	31 ± 5	32 ± 4	0.07
**Heart Weight (mg)**	129 ± 16	129 ± 10	0.97
**Tibial Length (cm)**	1.8 ± 0.0	1.8 ± 0.0	0.97
**HW/BW Ratio**	4.3 ± 0.4	4.0 ± 0.6*	**0.04**
**HW/TL Ratio**	7.2 ± 1.0	7.1 ± 0.5	0.90
**Spleen (mg)**	63 ± 8	64 ± 10	0.63
**Right Kidney (mg)**	158 ± 22	166 ± 20	0.13
**Liver (g)**	1.1 ± 0.1	1.2 ± 0.2*	**0.05**
**LV Hematocrit (%)**	37 ± 3	38 ± 2	0.77
**Saphenous Hematocrit (%)**	42 ± 4	43 ± 2	0.33
**Saphenous Hemoglobin (g/L)**	127 ± 12	134 ± 8	0.16

Significance was considered when *p* < 0.05 compared to EPO^fl/fl^ (determined by an unpaired, two-tailed t-test and shown using bolded values and "*").

### 3.2 EPO^Δ/Δ^ mice had greater voluntary wheel running capacity independent of changes in hematocrit and whole-body metabolism

Exposing mice to either a physiological or pathological challenge could reveal additional insight to the innate compensatory mechanisms. We therefore subjected mice to an exercise test, and considering there was no change in hematocrit, we hypothesized no difference would be observed between groups. Yet, voluntary wheel running across three consecutive days revealed EPO^Δ/Δ^ mice had increased running performance compared to control animals (Figure 2D). Since the difference in exercise capacity was not the result of erythropoiesis, we determined it could be linked to reported effects of EPO as a regulator of energy homeostasis as it increases metabolic activity, cellular respiratory capacity, and oxygen utilization shown previously in transgenic mice (Wang et al., 2013). However, using whole-body indirect calorimetry (CLAMS setup), our data showed there were no differences in VO_2_ (Figures 2E, F), VCO_2_ (Figures 2G, H), RER (Figures 2I, J), or total energy expenditure between groups (Figure 2K, L). We cannot exclude the possibility for differences in myocardial oxygen consumption at the level of the skeletal muscle by these data alone. It is plausible that EPO^Δ/Δ^ mice were more efficient at extracting oxygen for mitochondrial cellular respiration (i.e., ATP production). Indeed, positive hypertrophic cardiac remodeling might account for the heightened exercise tolerance due to increased cardiac output.

### 3.3 EPO^Δ/Δ^ mice demonstrated concentric cellular hypertrophy

**FIGURE 3:**
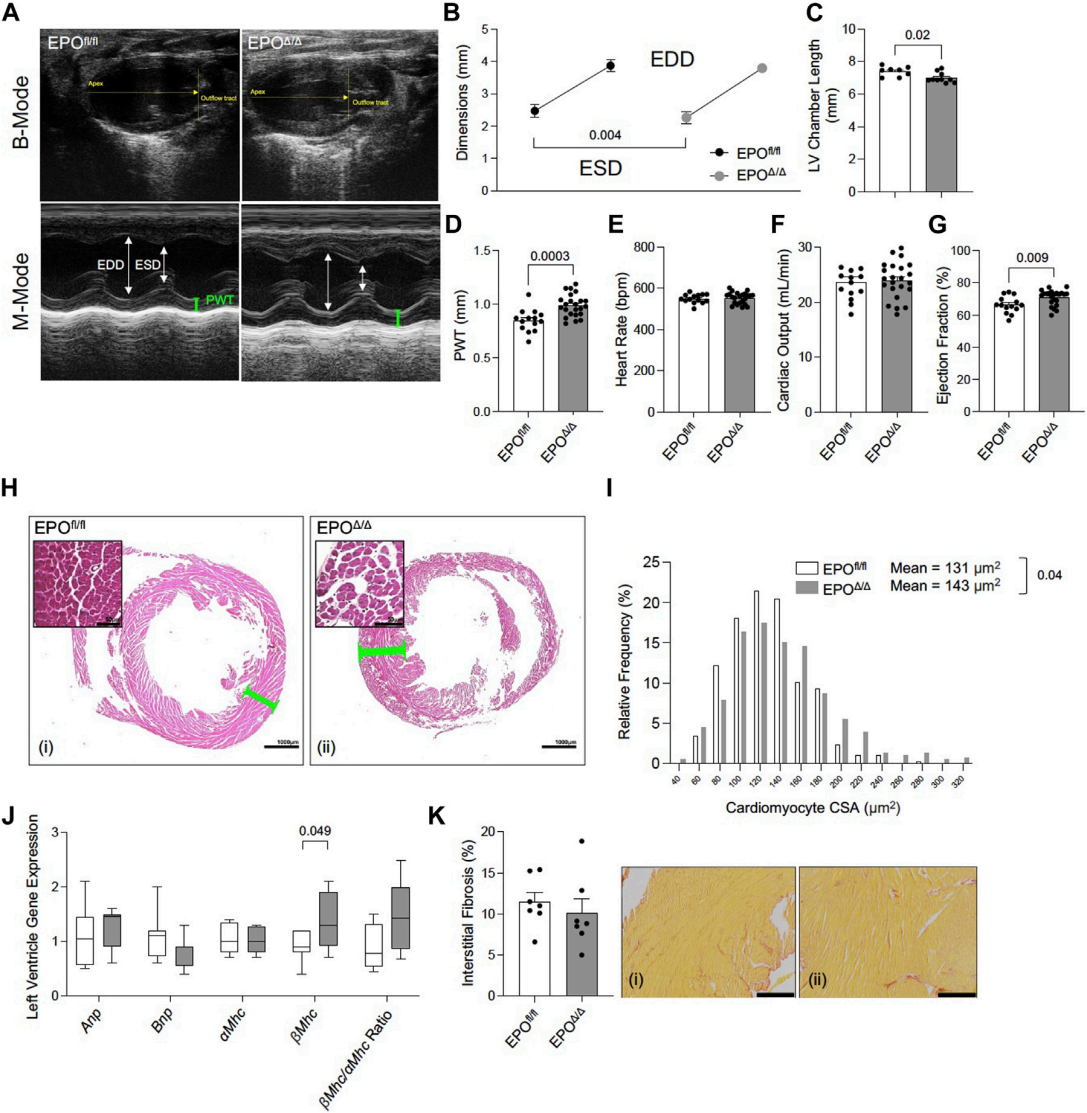
EPO^Δ/Δ^ mice exhibited compensatory changes in cardiac structure and systolic function by echocardiography. **(A)** Representative B-Mode (top) and M-Mode (bottom) tracings from EPO^fl/fl^ and EPO^Δ/Δ^ mice. Echocardiography measurements included **(B)** end systolic dimension (ESD) and end diastolic dimension (EDD), **(C)** left ventricle (LV) chamber length, **(D)** posterior wall thickness (PWT), **(E)** heart rate, **(F)** cardiac output, **(G)** ejection fraction. **(H)** Perfusion fixed hearts (representative hematoxylin and eosin histological images from (i) EPO^fl/fl^ and (ii) EPO^Δ/Δ^ hearts–scale bars represent 50µm and 1000 µm) were used to measure differences in **(I)** cardiomyocyte cross-sectional area (CSA). **(J)** Expression of fetal genes (atrial natriuretic peptide (*Anp*), brain natriuretic peptide (*Bnp*), alpha myosin heavy chain (*αMhc*), beta myosin heavy chain (*βMhc*), and *βMhc/αMhc* ratio) was quantified. **(K)** Quantification of interstitial fibrosis (%) from (i) EPO^fl/fl^ and (ii) EPO^Δ/Δ^ hearts stained with Picrosirius Red. Scale bar represent 216 µm. An unpaired, two-tailed t-test was used to detect differences. Data are expressed as mean ± SEM (for echocardiography, CSA, and interstitial fibrosis) or mean ± SD (qPCR). Data were considered significant when *p* < 0.05.

Prior work showed that constitutive deletion of cardiomyocyte-specific EPO during embryogenesis causes early hypoplasia, and eventually, cardiac *Epo* over-expression led to hypertrophy, with no change in overall cardiac mass in adult hearts (Allwood et al., 2024). We sought to explore the physiological significance of cardiomyocyte deleted *Epo* in adult mice, independent of cardiogenesis. By echocardiography, there was a significant decrease in end systolic dimension (ESD) and a trending reduction in end diastolic dimension in EPO^Δ/Δ^ mice (EDD, Figures 3A, B; Table 3). The left ventricle chamber length was decreased (Figures 3A, C) and posterior wall thickness was increased (Figures 3A, D). While heart rate (Figure 3E) and cardiac output (Figure 3F) were unchanged, EPO^Δ/Δ^ mice had greater ejection fractions (Figure 3G), suggesting systolic function was being augmented. Together, these parameters indicated a concentric hypertrophy phenotype in the EPO^Δ/Δ^ mice. To confirm these findings, we evaluated histologically cardiomyocytes in perfusion-fixed hearts (Figure 3H), identifying a significant increase in EPO^Δ/Δ^ cross-sectional area compared to control (Figure 3I). These data combined suggest that as cells of the left ventricle wall widened, the length of the hearts shortened, resulting in no difference in global heart mass.

**TABLE 3:** *In vivo* cardiac assessment using echocardiography and invasive hemodynamics. LVP, left ventricle peak pressure; LV EDP, left ventricle end diastolic pressure. Data is presented as mean ± SEM.

Echocardiography	EPO^fl/fl^ (n = 14)	EPO^Δ/Δ^ (n = 23)	*p*-value
M-Mode			
**Heart Rate (bpm)**	550 ± 6	553 ± 6	0.72
**Dimension**_**Systole**_ **(mm)**	2.5 ± 0.1	2.3 ± 0.0*	**0.004**
**Dimension**_**Diastole**_ **(mm)**	3.9 ± 0.0	3.8 ± 0.0	0.24
**Volume**_**Systole**_ **(uL)**	22 ± 1	18 ± 1*	**0.01**
**Volume**_**Diastole**_ **(uL)**	65 ± 2	62 ± 2	0.25
**Stroke Volume (uL)**	43 ± 2	44 ± 1	0.70
**Ejection Fraction (%)**	66 ± 1	71 ± 1*	**0.009**
**Fractional Shortening (%)**	36 ± 1	40 ± 1*	**0.009**
**Cardiac Output (mL/min)**	24 ± 1	24 ± 1	0.78
**Wall Thickness**_**Diastole**_ **(mm)**	0.85 ± 0.0	0.99 ± 0.0*	**0.0003**

	EPO^fl/fl^ (n = 8)	EPO^Δ/Δ^ (n = 10)	*p*-value
B-Mode			
**LV Chamber Length (mm)**	7.4 ± 0.1	7.0 ± 0.1*	**0.02**

Left Ventricle Hemodynamics	EPO^fl/fl^ (n = 14)	EPO^Δ/Δ^ (n = 18)	*p*-value
**Heart Rate (bpm)**	538 ± 7	543 ± 6	0.97
**LVP (mmHg)**	103 ± 2	108 ± 1*	**0.005**
**LV EDP (mmHg)**	6 ± 1	6 ± 0	0.94
**Systolic Pressure (mmHg)**	98 ± 2	102 ± 2	0.11
**Diastole Pressure (mmHg)**	63 ± 2	67 ± 1	0.20
**Mean Arterial Pressure (mmHg)**	75 ± 2	79 ± 1	0.15
**dP/dt max (mmHg/s)**	9840 ± 155	11,387 ± 189*	**<0.0001**
**dP/dt min (mmHg/s)**	−9337 ± 206	−10336 ± 264*	**0.005**
**dP/dt @ LVP 40 (mmHg/s)**	9297 ± 152	10,391 ± 178*	**<0.0001**
**Tau Logistic (ms)**	4.3 ± 0.2	4.1 ± 0.1	0.35

Significance was considered when *p* < 0.05 compared to EPO^fl/fl^ (determined by an unpaired, two-tailed t-test and shown using bolded values and "*").

Both pathological (e.g., hypertension) and physiological (e.g., exercise) conditions stimulate cellular and gross ventricular hypertrophy with inverse relationships to long-term health (Schoenfeld et al., 1998). However, a hallmark feature of pathological remodeling is the re-expression of certain fetal genes (Schoenfeld et al., 1998). Therefore, we quantified relative RNA levels of *Anp*, *Bnp*, *α-Mhc*, *ß-Mhc*, and the ratio of *ß-Mhc/α-Mhc* between groups by qPCR. No differences amongst *Anp*, *Bnp*, *α-Mhc* expression, or the ratio of *ß-Mhc/α-Mhc* (Figure 3J) were seen though *ß-Mhc* RNA was increased in EPO^Δ/Δ^ mice. An incipient cause of *ß-Mhc* re-expression during cardiac hypertrophy and normal aging is linked to elevated fibrosis (Pandya et al., 2006). Accordingly, we quantified interstitial fibrosis of the left ventricle between groups (Figures 3Ki,ii), finding no difference. EPO^Δ/Δ^ mice had compensatory cardiac function (i.e., increased ejection fraction) and the EPO^Δ/Δ^ phenotype is inconsistent with pathological remodeling. Nonetheless, we reconciled this line of inquiry with a comprehensive assessment of cardiac function by invasive hemodynamics.

### 3.4 EPO^Δ/Δ^ mice have heightened cardiac function *in vivo*

**FIGURE 4:**
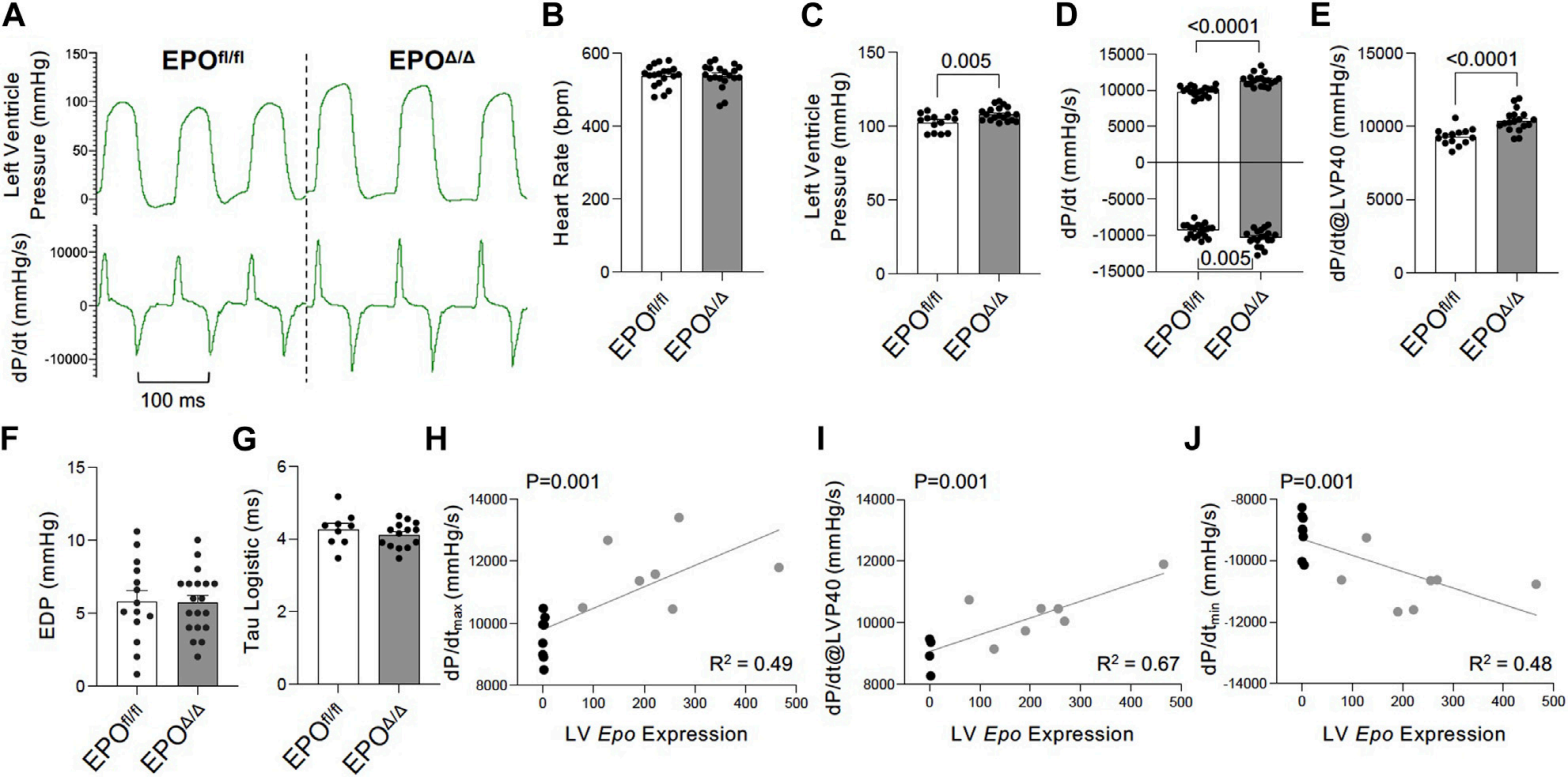
Improved inotropic and lusitropic cardiac function was observed *in vivo* by invasive hemodynamics in EPO^Δ/Δ^ compared to EPO^fl/fl^ mice. **(A)** Representative left ventricle pressure (LVP, top) and dP/dt_max_ and dP/dt_min_ (bottom) tracings from EPO^fl/fl^ and EPO^Δ/Δ^ mice. **(B)** Heart rate (bpm), **(C)** left ventricular systolic pressure (mmHg), **(D)** dP/dt_max_ and dP/dt_min_ (mmHg/s), **(E)** dP/dt@LVP40 (mmHg/s), **(F)** end diastolic pressure (EDP), and **(G)** Tau logistic (ms). Simple linear regression correlation analyses were run comparing *Epo* RNA expression to **(H)** dP/dt_max_, **(I)** dP/dt@LVP40, and **(J)** dP/dt_min_ values and detected a positive correlation (*p* = 0.0014 for each) with moderate to strong goodness-of-fit values (R^2^ = 0.49, 0.67, 0.48, respectively). An unpaired two-tailed t-test was used to detect differences. Data are expressed as mean ± SEM and were considered significant when *p* < 0.05.

To gain a deeper understanding of the functional role of cardiac *Epo* overexpression and concentric cellular hypertrophy on cardiac function in our model, invasive hemodynamic analyses were performed (Figure 4A; Table 3). There were no differences in heart rate (Figure 4B). In terms of systolic function, EPO^Δ/Δ^ mice demonstrated elevated left ventricular systolic pressures (LVP, Figure 4C) and an increase in cardiac contractility (dP/dt_max_, dP/dt@LVP40, Figures 4D, E). To assess diastolic function, a multi-parameter approach was used (Ogilvie et al., 2020). For this reason, we reported indices of both relaxation and compliance: dP/dt_min_ (active relaxation phase), Tau Logistic (active relaxation phase), and end diastolic pressure (EDP, passive filling phase). dP/dt_min_ was significantly improved (Figure 4D), though this was not reflected by differences in EDP (Figure 4F) or Tau Logistic (Figure 4G). Taken together, both systolic and diastolic function were superior in the EPO^Δ/Δ^ mice, suggesting endogenous cardiac EPO production was associated with better cardiac function. Supraphysiological levels of exogenous rhEPO confer inotropic and lusitropic effects *ex vivo*, therefore we were interested in the correlation between the levels of upregulated endogenous *Epo* RNA and these key hemodynamic parameters recorded *in vivo*. Simple linear regression analyses demonstrated a strong positive relationship between *Epo* RNA expression and dP/dt_max_, dP/dt_min_, and dP/dt@LVP40, with goodness-of-fits of R^2^ = 0.49, 0.48, and 0.67, respectively (Figures 4H–J). Therefore, our results indicate that in mice with higher endogenous cardiac *Epo* expression, inotropic and lusitropic function are greater.

### 3.5 Cardiac-derived EPO has inotropic, lusitropic, chronotropic, and cardioprotective benefits in an *ex vivo* isolated heart preparation

**FIGURE 5:**
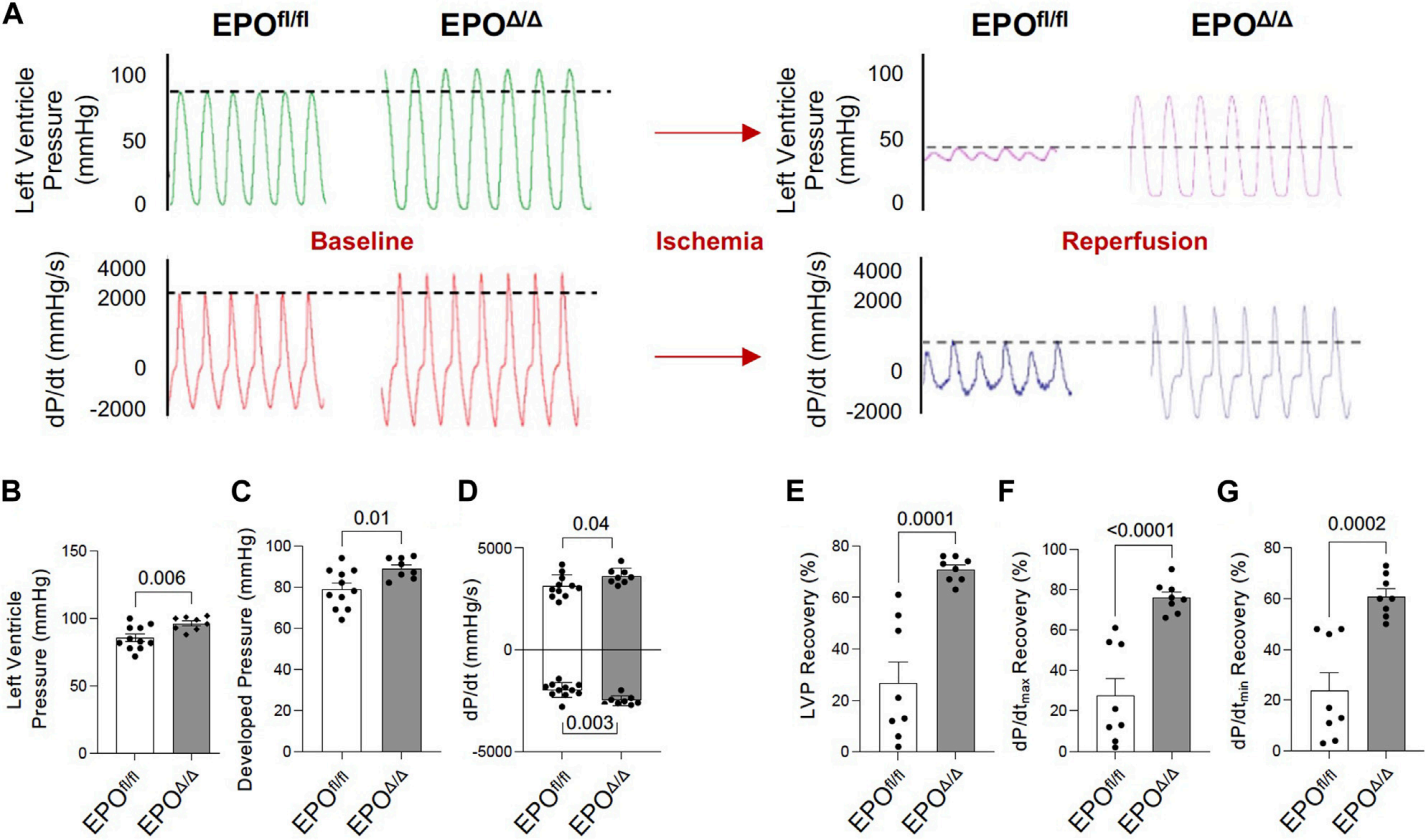
EPO^Δ/Δ^ mice demonstrated greater inotropic and lusitropic cardiac function by the *ex vivo* Langendorff preparation, accompanied by superior cytoprotective abilities post-ischemia/reperfusion. **(A)** Representative isolated heart (Langendorff) function tracings at baseline (red and green) and post-ischemia reperfusion (pink and purple) for EPO^fl/fl^ and EPO^Δ/Δ^ mice. Baseline measurements included **(B)** left ventricle systolic pressure (mmHg), **(C)** developed pressure (mmHg), **(D)** dP/dt_max_ and min (mmHg/s). Baseline was followed by global no-flow ischemia and subsequent reperfusion, generating data for **(E)** % left ventricular pressure (LVP) recovery, **(F)** % dP/dt_max_ recovery, and **(G)** % dP/dt_min_ recovery. An unpaired two-tailed t-test was used to detect differences. Data are expressed as mean ± SEM and were considered significant when *p* < 0.05.

The *in vivo* assessment of cardiac function carries limitations–the data collected are dependent on preload, afterload, and heart rate. Conversely, the Langendorff preparation (i.e., *ex vivo*) allows for the isolated evaluation of systolic and diastolic cardiac function at a healthy baseline or after ischemic insult under controlled conditions without the influence of systemic neurohormonal or immunological factors. Since exogenous rhEPO has shown cardioprotective effects *in vivo*, investigating the physiological autocrine and/or paracrine effects of endogenous cardiac EPO on the heart was of particular interest. The EPO^Δ/Δ^ group demonstrated a significant increase in baseline LVP (Figures 5A, B (top left)), developed pressure (Figure 5C), and dP/dt_max_ and dP/dt_min_ (Figure 5A (bottom left), D). While there were no significant differences in EDP or paced heart rate as they were manually set and controlled, intrinsic heart rate prior to pacing was elevated in EPO^Δ/Δ^ mice (Table 4). After baseline recordings, mice were subjected to 25 min of global, no-flow ischemia, followed by 45 min of reperfusion (Figure 5A (right)). EPO^Δ/Δ^ mice displayed better recovery post-ischemia: 71% of LVP (Figure 5E), 76% of dP/dt_max_, (Figure 5F), and 61% of dP/dt_min_ (Figure 5G), whereas the EPO^fl/fl^ mice recovered only 27% of LVP (Figure 5E), 28% of dP/dt_max_ (Figure 5F), and 25% of dP/dt_min_ (Figure 5G). Therefore, independent of heart rate, afterload, preload, and whole-body neurohormonal influence, hearts overexpressing endogenous cardiac EPO also displayed positive inotropic, lusitropic, chronotropic, and cardioprotective qualities. Having established the physiological impact of hyper-compensated cardiac *Epo*, we aimed to clarify some of the factors involved in regulating this phenomenon.

**TABLE 4:** *Ex vivo* cardiac assessment using Langendorff isolated heart preparation. LVP, left ventricle systolic pressure; LV EDP, left ventricle end diastolic pressure. Data is presented as mean ± SEM.

	EPO^fl/fl^ (n = 8)	EPO^Δ/Δ^ (n = 8)	*p*-value
**Intrinsic Heart Rate (bpm)**	203 ± 25	342 ± 26*	**0.002**
**LVP (mmHg)**	86 ± 3	96 ± 2*	**0.006**
**LV EDP (mmHg)**	7 ± 1	7 ± 0	0.30
**Developed Pressure (mmHg)**	79 ± 3	89 ± 2*	**0.014**
**dP/dt max (mmHg/s)**	3128 ± 166	3626 ± 135*	**0.04**
**dP/dt min (mmHg/s)**	−1978 ± 108	−2488 ± 82*	**0.003**
**% Recovery LVP**	27 ± 8	71 ± 2*	**0.0001**
**% Recovery dP/dt max**	28 ± 9	76 ± 3*	**<0.0001**
**% Recovery dP/dt min**	24 ± 7	61 ± 3*	**0.0002**

Significance was considered when *p* < 0.05 compared to EPO^fl/fl^ (determined by an unpaired, two-tailed t-test and shown using bolded values and "*").

### 3.6 Marked overexpression of left ventricular *Epo* in EPO^Δ/Δ^ mice was HIF2α-independent

**FIGURE 6:**
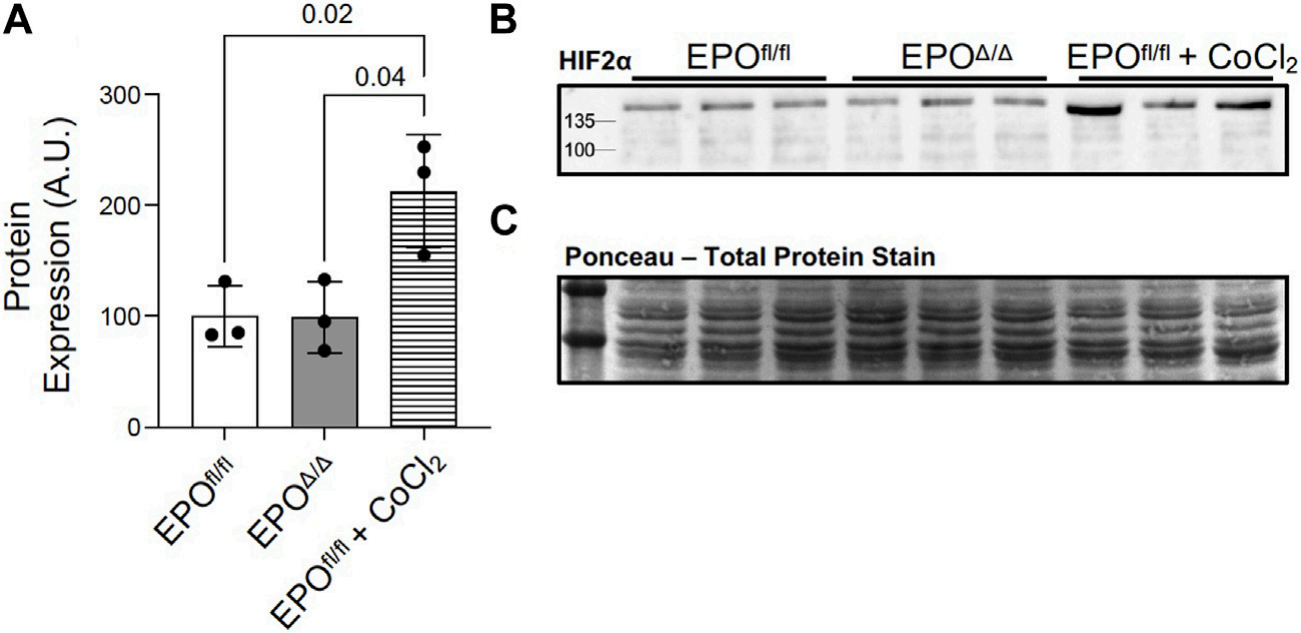
Marked overexpression of left ventricular *Epo* in EPO^Δ/Δ^ mice was HIF2α-independent. **(A)** Western blot permitted the quantification of HIF-2α protein from nuclear extracts of left ventricle tissue of EPO^fl/fl^, EPO^Δ/Δ^, and EPO^fl/fl^ mice treated with cobalt chloride (CoCl_2_) at 3 timepoints–3h post-injection, 1.5h post-injection, and 45min post-injection. **(B)** Representative western blot of HIF-2α and **(C)** ponceau stain, which demonstrates equal loading (20 µg per well). A one-way ANOVA followed by a Tukey’s post-hoc test was used to detect differences amongst the three groups. Data are expressed as mean ± SD and were considered significant when *p* < 0.05.

HIF-1α and HIF-2α are both primary transcription factors for regulating the hypoxic response. However, in terms of the transactivation of the *Epo* promoter, HIF-2α, and not HIF-1α, is the primary regulator in the adult kidney, liver, and brain (Warnecke et al., 2004; Gruber et al., 2007; Rankin et al., 2007; Yamashita et al., 2008; Yeo et al., 2008; Wang et al., 2015; Urrutia et al., 2016; Ghosh et al., 2021). Although there was no overt indication of hypoxia in this model (i.e., no differences in hematocrit or renal EPO expression), we aimed to rule out the canonical PHD2/VHL/HIF2α axis as the mechanism for mediating cardiac overexpression of *Epo* in EPO^Δ/Δ^ mice. By immunoblotting, we showed no difference in the HIF-2α protein levels in left ventricular nuclear extracts of EPO^fl/fl^ and EPO^Δ/Δ^ mice (Figures 6A–C). This finding confirmed the overexpression of left ventricular *Epo* in EPO^Δ/Δ^ mice was HIF2α-independent. Considering the unexpected nature of our findings, we wanted to conclusively exclude hypoxia as a stimulus for cardiac overexpression of *Epo* by investigating HIF1α-specific downstream target genes.

### 3.7 Overexpression of cardiac *Epo* in EPO^Δ/Δ^ mice was accompanied by elevated *Vegfb* and *Vegfr1* gene expression

**FIGURE 7:**
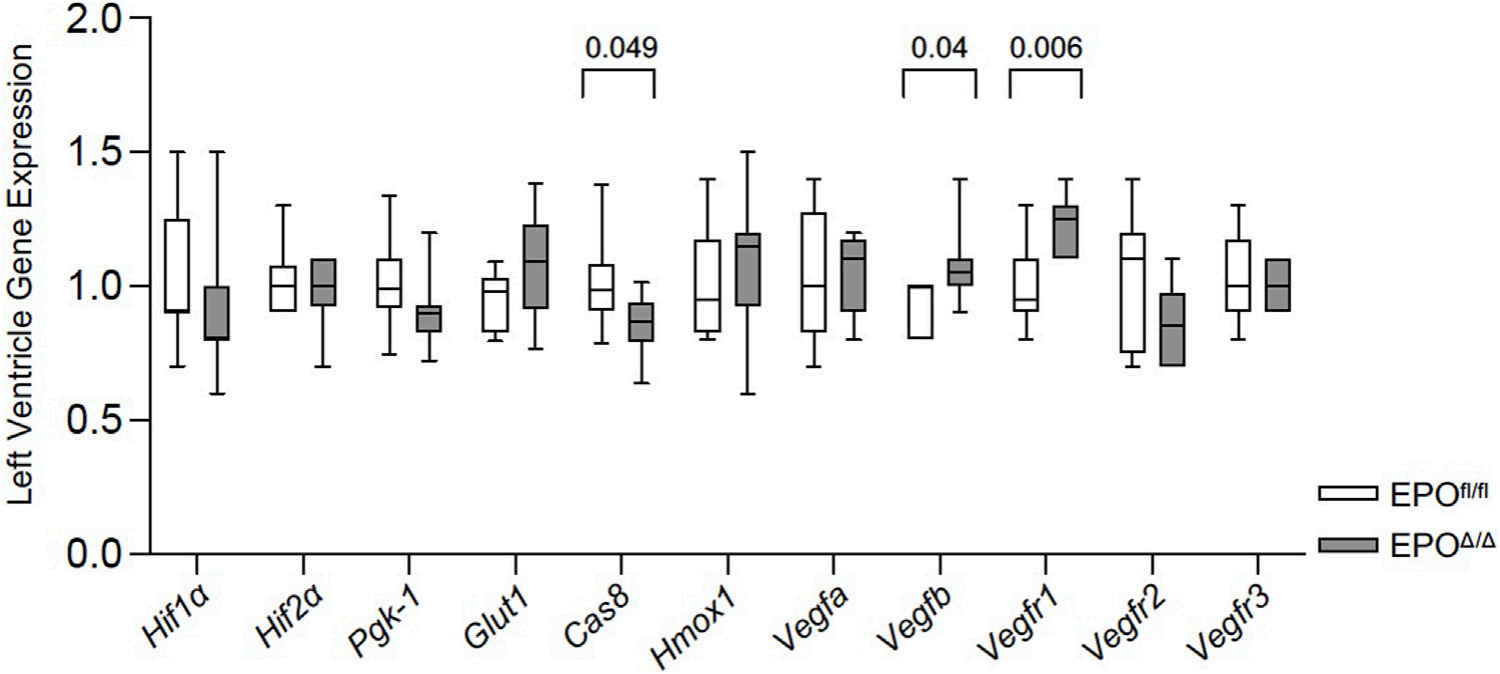
*Vegfb* and *Vegfr1* RNA expression were upregulated in the left ventricle of EPO^Δ/Δ^ mice. An unpaired two-tailed t-test was used to detect differences between EPO^fl/fl^ and EPO^Δ/Δ^ for individual genes of interest using qPCR. Data are expressed as mean ± SD and were considered significant when *p* < 0.05.

Under hypoxic conditions, HIF-1α escapes oxygen-dependent degradation, translocates to the nucleus, and complexes with HIF-1β (Wang et al., 1995). Working in concert with hepatocyte nuclear factor 4 (HNF-4) (Galson et al., 1995; Huang et al., 1997; Jelkmann, 2007) and the transcriptional co-activators, p300 and cAMP response element (CREB)-binding protein (Firth et al., 1995; Bunn et al., 1998), the HIF-1 complex binds the hypoxia response element to initiate the transcription of >500 downstream target genes (Mole et al., 2009), including *Vegfa* (Forsythe et al., 1996), *Glut1* (Iyer et al., 1998), *Ho-1* (Lee et al., 1997), *Pgk-1* (Semenzas et al., 1994; Li et al., 1996a), *Cas 8* (Zhao et al., 2018). Therefore, to rule out hypoxia and the involvement of HIF-1α in regulating cardiac *Epo* overexpression, we examined the expression levels of select HIF1α-specific target genes (e.g., *Vegfa*, *Glut1*, *Hmox-1*, *Pgk-1*, *Cas 8*) by qPCR. We measured no differences in their gene expression, and even downregulation of *Cas 8* in EPO^Δ/Δ^ mice (Figure 7). This indicated the upstream regulation of *Epo* in the EPO^Δ/Δ^ mice by hypoxia and HIF-1α was unlikely. *Epo* is also regulated by HIF-independent mechanisms in a tissue-specific manner (Yasuda et al., 1998; Tam et al., 2006). One study demonstrates potent inhibition of VEGF induces hepatic synthesis of *Epo* and subsequent erythropoiesis through a HIF1α-independent mechanism (Tam et al., 2006). Therefore, we investigated the hypoxia-independent VEGF isoform, *Vegfb*, and its receptor, *Vegfr1*, by qPCR analyses. Interestingly, both genes were significantly upregulated in EPO^Δ/Δ^ mice (Figure 7). Co-upregulation of endothelial-derived EPO and whole-heart *Vegfb* and *Vegfr1* suggested a complex cellular interaction was governing this novel physiological concentric hypertrophy and cardioprotective phenotype. To identify a relationship between cardiac EPO and VEGF, we used the FDA-approved VEGF-specific tyrosine kinase inhibitor, axitinib, to interrupt VEGF signaling and observe the corresponding impact on *Epo* RNA levels in the heart.

### 3.8 Axitinib disrupted the VEGF-VEGFR-dependent crosstalk between endothelial cells and cardiomyocytes, which further upregulated EPO expression in mice

**FIGURE 8:**
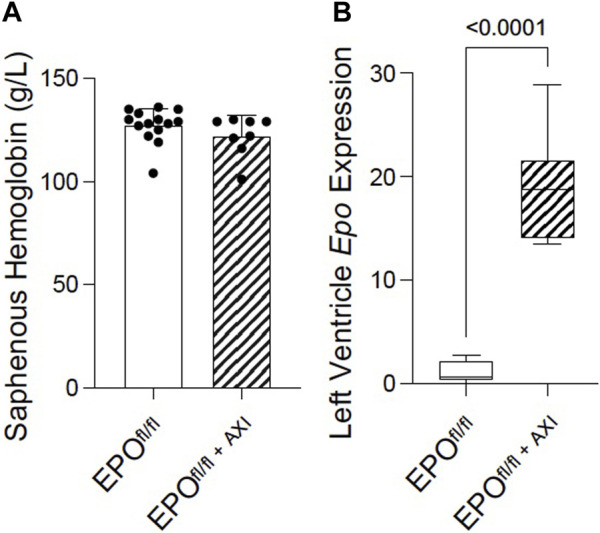
Pan-VEGFR inhibition via 8 days of axitinib treatment (i.p.) significantly upregulated left ventricular *Epo* RNA expression in EPO^fl/fl^ mice independent of erythropoiesis. **(A)** Saphenous hemoglobin levels and **(B)** left ventricle *Epo* RNA expression by qPCR in EPO^fl/fl^ compared to EPO^fl/fl+AXI^ mice. Data are expressed as mean ± SD and were considered significant when *p* < 0.05 as determined by unpaired, two-tailed t-test.

Several independent labs show active systemic crosstalk between EPO-EPOR and VEGF-VEGFR signal transduction pathways. rhEPO increases *Vegf* expression (Ribatti et al., 1999; Westenbrink et al., 2008; Lu et al., 2012; Oztas et al., 2020) resulting in angiogenesis and attenuated interstitial fibrosis, while VEGF-VEGFR blockade induces non-renal *Epo* expression (Tam et al., 2006) and erythropoiesis (Tam et al., 2006; Johnson et al., 2017). Whether these pathways interact similarly in the heart by endogenous forms of EPO and VEGF, is unknown. Therefore, to improve our understanding of the interplay between VEGF-VEGFR and EPO-EPOR in the heart, we interrupted VEGF-VEGFR signaling by inhibition using axitinib (Ma and Waxman, 2009) in EPO^fl/fl^ mice. Preclinically, in a mouse model of prostate cancer, 24 days of axitinib treatment effectively arrests tumour growth by supressing tumour patency (i.e., blood perfusion) without disrupting blood perfusion to normal tissues (e.g., heart, kidney, liver, lung, muscle) (Ma and Waxman, 2009). In our study, axitinib was well-tolerated, and mice did not display any overt signs of distress during, or after, treatment. Short-term axitinib treatment did not elicit changes in erythropoiesis, as determined by no differences detected in hemoglobin levels measured between EPO^fl/fl^ and EPO^fl/fl+AXI^ groups (Figure 8A). When VEGF signaling was inhibited, cardiac expression of *Epo* RNA was significantly elevated by qPCR (Figure 8B). However, by invasive hemodynamics, we observed blunted heart rates and reduced left ventricle relaxation in axitinib-treated mice (Supplementary Table S2). These data were expected since tyrosine kinase inhibitors affect the cardiac conduction system, causing bradycardia and QTc prolongation (Kloth et al., 2015; Shopp et al., 2022), and are known to have cardiotoxic effects (Force and Kolaja, 2011; Dobbin et al., 2021). Our findings indicated cardiac *Epo* upregulation following VEGF inhibition does not rescue hearts from suspected axitinib-related bradycardia and cardiotoxicity. Importantly, however, we revealed the existence of a previously unrecognized link between VEGF and EPO in the heart. Future work is needed to fully define how the cardiac EPO-EPOR and VEGF-VEGFR axes are coordinated.

## 4 Discussion

Here we show that targeted deletion of the *Epo* gene in the adult cardiomyocyte results in compensatory overexpression by cardiac endothelial cells by HIF2α-independent conditions. The presence of excess *Epo* in the heart translated to notable changes in cardiac structure, and improvements in function and cytoprotection. Hyperproduction of *Epo* by the heart did not affect systemic erythropoiesis, suggesting EPO only acted locally via paracrine signaling between cardiomyocytes and endothelial cells. Pharmacological interruption of VEGF-VEGFR using the tyrosine kinase inhibitor, axitinib, allowed us to study how cardiac *Epo* expression could be modulated in the absence of VEGF signaling. Indeed, cardiac *Epo* RNA levels were upregulated in axitinib-treated mice. These data suggested a dynamic and interactive crosstalk between cardiomyocytes and endothelial cells involving the VEGF-VEGFR pathway that regulate the upstream production and subsequent physiological effects of cardiac EPO under hypoxia-independent conditions. Taken together, in the adult mouse heart, endothelial EPO is an important regulator of cell infrastructure, cardiac contractility, and ischemia-reperfusion susceptibility, such that excess *Epo* is associated with superior cardiac function directly independent of systemic red blood cell production.

Recent studies establish EPO as a pleiotropic cytokine. However, interpretation of these data does not always consider the form of EPO in question–exogenous vs. endogenous. rhEPO is differentially glycosylated compared to endogenous EPO (Rush et al., 1995; Skibeli et al., 2001; Lasne et al., 2002) and the oligo branching patterns and sialic acid content modify the pharmacodynamics and biological activity *in vivo* (i.e., stability and receptor binding) (Weikert et al., 1999; Skibeli et al., 2001). Therefore, high dose rhEPO may facilitate non-specific receptor binding, signal transduction, and physiological outcomes that do not apply to endogenous EPO. According to three *ex vivo* studies (Kaygisiz et al., 2006; Piuhola et al., 2008; Hefer et al., 2012), exogenous rhEPO is reportedly inotropic. The changes in contractility and relaxation are attributed to cAMP (Kaygisiz et al., 2006), increased calcium transients (rate and magnitude) and myofilament function via PI3-K and PKCε (Hefer et al., 2012), and endothelin-1 signaling (Piuhola et al., 2008). In another *ex vivo* study, hypoxia induces high endogenous plasma EPO levels, which correlate to increased atrial contraction (Sterin-Borda et al., 2003). Collectively, these studies suggest a positive primary effect of EPO on myocardial inotropic function.

Until now, the interpretation and application of these findings to an *in vivo* endogenous EPO system was limited. Therefore, using both *ex vivo* (i.e., Langendorff isolated hearts) and *in vivo* (i.e., invasive hemodynamics) preparations, our data revealed the improved cardiac functional effects conferred by endogenous EPO in EPO^Δ/Δ^ mice for the first time. It is plausible endogenous EPO improved calcium handling by increasing cAMP-dependent protein kinase A (PKA) phosphorylation of phospholamban (PLB) (Simmerman and Jones, 1998), which stimulated subsequent sarcoplasmic reticulum calcium uptake (Kranias et al., 1985) and release by the ryanodine receptors (RyR) (Valdivia et al., 1995). Indeed, calcium transient assays and western blotting of key proteins (i.e., PLB, RyR) may clarify this line of inquiry. Alternatively, greater cardiac function in EPO overexpressing mice could be attributed to cardiomyocyte hypertrophy (i.e., CSA ∝ force output) (An et al., 1991). Investigating additional biochemical parameters (e.g., nitric oxide, endothelial or neuronal nitric oxide synthase, TGF-β, cyclic GMP) could clarify the mechanism(s) underlying this work, albeit they remain unclear. Since both cardiomyocytes (Wright et al., 2004) and endothelial cells (Anagnostou et al., 1994; Beleslin-Cokic et al., 2004; Yang et al., 2014) express the *Epor*, the EPO-mediated contractile effects could be the result of total activation of one, or both, cell receptors (Sterin-Borda et al., 2003). Thus, studies relying on transgenic knockout mice, radiolabeled EPO-receptor binding assays, immunoprecipitation, and western blotting, will ultimately improve our knowledge of the non-canonical EPO receptor activation mechanisms that govern EPO-mediated improvements on cardiac function.

We are the first to report that targeted deletion of *Epo* from the cardiomyocyte, both in the embryonic (Allwood et al., 2024) and now adult hearts (data presented here), results in a hyper-compensated response by the cardiac endothelial cell. This phenomenon suggests a critical physiological role for cardiac EPO, which must be conserved. The findings presented here were previously unrecognized. Using the same EPO^fl/fl^ mice, Zeigler et al. characterized a conditional EPO-deficient model for chronic kidney disease (CKD) by tamoxifen-induced whole-body *Epo* knockout in the adult mouse (Zeigler et al., 2010). Considering 80%–95% efficiency of Cre recombination (Sohal et al., 2001; Hsieh et al., 2007; Bersell et al., 2013), their model achieves near ubiquitous *Epo* knockout from all cell types within the heart (Zeigler et al., 2010). Since the focus of their study was to establish the CKD model and prove EPO-deficient mice still participate in stress-induced erythropoiesis, no further physiological investigations were performed on the heart. Therefore, our study partially resolves this limitation and confirms a critical role for cardioendothelial derived *Epo* in modulating cardiac morphology, function, and susceptibility to ischemia. However, the complex cellular interplay we observed may not be limited to the cardiomyocytes and endothelial cells. For instance, decades of research indicate renal EPO synthesis is the sum of multiple EPO-producing cells (e.g., cells of the interstitial cortex and outer medulla (Maxwell et al., 1993), proximal convoluted tubule cells (Loya et al., 1994; Haidar et al., 1997), and peritubular fibroblasts (Koury et al., 1988; Lacombe et al., 1988))–therefore, it is plausible the novel regulatory network presented here extends to and includes other cardiac cells (e.g., smooth muscle cells, cardiac progenitors, monocytes/macrophages). While cardiac fibroblast cell isolation revealed no detectable *Epo* mRNA signal from either group (data not shown), other resident cardiac cells capable of *Epo* production in the heart could be identified using single cell RNA sequencing, fluorescent *in situ* hybridization, or *Epo* reporter mice should be used (Kragesteen et al., 2023). These findings would greatly inform the relevance of non-erythropoietic non-renal EPO, and open new avenues for EPO regulation to be explored.

**FIGURE 9:**
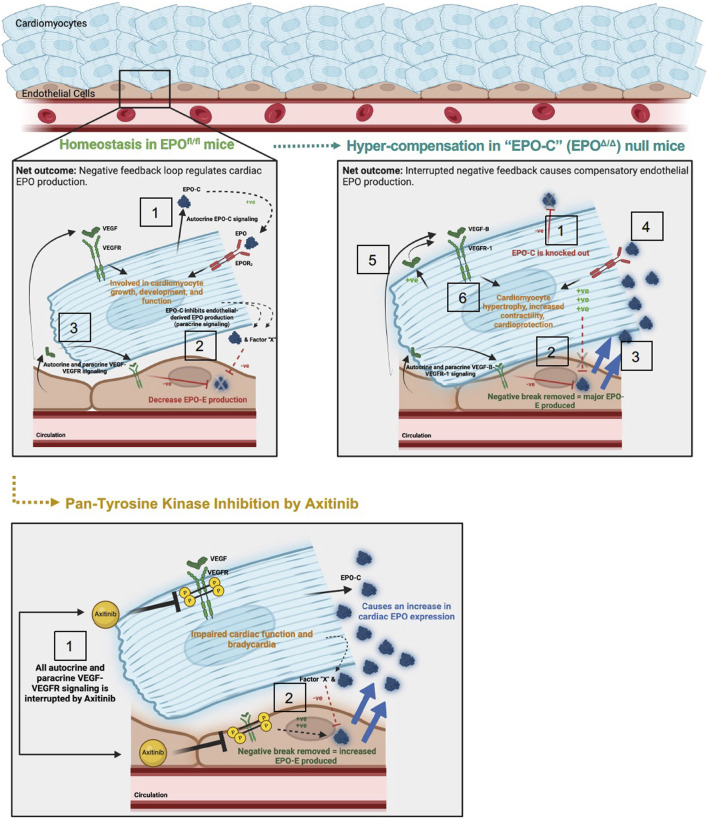
Schematic of proposed mechanism: cardiac structure and function rely on the homeostatic interaction between EPO-EPOR and VEGF-VEGFR signaling. Top left panel: (1) In adult wildtype mice (EPO^fl/fl^), the cardiomyocyte produces low levels of *Epo* at baseline (Figure 1). (2) Cardiomyocyte derived EPO (“EPO-C″) represses endothelial cell EPO (“EPO-E″) production in a paracrine fashion. (3) VEGF elicits paracrine stimulation of VEGFR on neighboring endothelial cells and cardiomyocytes, and activation of this pathway inhibits EPO-E production. The net outcome of these stimuli is reciprocal endothelial repression of EPO production. Top right panel: (1) In EPO-C null mice (EPO^Δ/Δ^) EPO is successfully knocked out of the cardiomyocyte. (2) The lack of EPO produced by the cardiomyocytes releases the inhibition of EPO-EPOR by the endothelial cell in a paracrine fashion. (3) This leads to overproduction of EPO by the endothelial cell. (4) The overproduction of EPO positively feeds back and binds the EPOR_2_ located on the cardiomyocyte (Wright et al., 2004). EPO binding to the EPOR_2_ on the cardiomyocyte may induce cardiomyocyte-production of VEGF. (5) Increased VEGF-B-VEGFR-1 signaling, along with (6) additional unknown intermediate factors, increase myocyte hypertrophy, contractile function, and cardioprotection (Karpanen et al., 2008b; Zentilin et al., 2010; Kivelä et al., 2014; Lal et al., 2017). Bottom panel: (1) Axitinib, a pan-tyrosine kinase inhibitor, prevents downstream VEGF-VEGFR signaling. (2) VEGF-induced inhibition of EPO is interrupted, resulting in an increase in EPO-E and EPO-C production. The net outcome of VEGF-VEGFR inhibition is impaired cardiac function and bradycardia, despite increased EPO production. The mechanisms underlying these physiological consequences require future work.

Cardiac structure and function are governed by complex cellular interplay, that when disturbed, can cause pathology (Yin et al., 2017). Given their proximity, non-myocytes (e.g., fibroblasts, endothelial cells) regulate cardiomyocyte growth and development either through direct cell-to-cell contact or by the release of paracrine factors (Long et al., 1991; Gray et al., 1998; Yin et al., 2017). Cardiomyocytes, the force-producing cells of the heart, are unique from non-myocytes in that they very rarely self-replicate (Long et al., 1991; Li et al., 1996b; Soonpaa et al., 1996; Poolman and Brooks, 1998). Therefore, should cardiomyocyte-derived EPO be critical for global heart function, it is reasonable to speculate there is a feedforward mechanism involving other cell type(s) to compensate for any loss (proposed mechanism presented in Figure 9). Mice null for either *Epo* or the *Epor* die due to vascular abnormalities and anemia (Wu et al., 1999; Kertesz et al., 2004; Allwood et al., 2024). The data presented here emphasize that cardiac *Epo* production is also imperative for homeostatic function in the adult heart and is thus regulated by complex paracrine mechanisms that allow for redundancy to ensure adequate paracrine EPO is ever-present. Interestingly, this is not the case for VEGF-A. Upon cardiomyocyte or endothelial cell specific *Vegfa* deletion, no other VEGF isoforms or neighbouring cell types will increase their expression to compensate (Giordano et al., 2001). In these models, a lack of paracrine support leads to profound detriments in vascular homeostasis and cardiac function. The presence of redundancy for one system (i.e., EPO) and not the other (i.e., VEGF), is perhaps surprising considering the parallels between these growth factors and their upstream regulation by the HIFs, but could be explained, at least in part, by the isoform and/or transcription factors involved.

Dynamic cellular crosstalk between cardiomyocytes and endothelial cells involving EPO and VEGF signaling is likely as both the ligands and receptors have been localized to each (i.e., on cardiomyocytes: *Epor* (Wright et al., 2004), *Vegfr* (Zentilin et al., 2010), *Epo* (Mengozzi et al., 2006; El Hasnaoui-Saadani et al., 2013), and *Vegf* (Giordano et al., 2001); on endothelial cells: *Epor*_*2*_ (Anagnostou et al., 1994; Beleslin-Cokic et al., 2004; Yang et al., 2014), *Vegfr* (Lee et al., 2007), *Epo* (Mengozzi et al., 2006; Allwood et al., 2024), and *Vegf* (Lee et al., 2007)). On a whole-heart level, endogenous inactivation of the EPO-EPOR system reduces cardiomyocyte *Vegf* production and angiogenesis resulting in worse cardiac function in a model of pressure-overload (Asaumi et al., 2007). When cardiomyocyte derived *Vegfa* is knocked out during development, the adult heart demonstrates hypovascularity, cardiac dysfunction, and hypoxia induced *Epo* expression (Lee et al., 2007). Therefore, crosstalk amongst the EPO and VEGF axis appears to modulate morphology, contraction, and protection within the heart. At the molecular level, we observed elevated *Epo* RNA in EPO^Δ/Δ^ mice alongside a significant increase in *Vegfb* gene expression (the hypoxia-independent form of VEGF predominantly found in the heart) and its receptor, *Vegfr1*. When VEGF-VEGFR signaling was neutralized in axitinib-treated mice, cardiac EPO expression (Figure 8B) was upregulated–a schematic representation of this new conceptual mechanism remains to be further defined (Figure 9). The uniform expression pattern of *Epo*, *Vegfb*, *Vegfr1* in the EPO^Δ/Δ^ heart may represent an intricate modulatory mechanism wherein VEGF-B indirectly drives cardiomyocyte hypertrophy, leading to better cardiac function, and lower susceptibility to ischemia-reperfusion injury (Shiojima et al., 2005; Tirziu et al., 2007; Karpanen et al., 2008a; Karpanen et al., 2008b; Zentilin et al., 2010; Kivelä et al., 2014; Lal et al., 2017; Lal et al., 2018). All in all, within the heart, the EPO-EPOR/VEGF-VEGFR axis exists to support cardiac homeostasis.

Hypoxia induced *Epo* synthesis in the kidney, liver, and brain is modulated by HIF-2α (Warnecke et al., 2004; Gruber et al., 2007; Rankin et al., 2007; Yamashita et al., 2008; Wang et al., 2015; Urrutia et al., 2016). However, the transcription factor responsible for cardiac *Epo* expression under hypoxic conditions (Chu et al., 2007; El Hasnaoui-Saadani et al., 2013; Deji et al., 2015) has received less attention. In one study, acute *Vhl* inactivation induces cardiac *Epo* production in a HIF1α-dependent manner. However, HIF-2α was not investigated under these conditions, therefore we cannot rule out its involvement in mediating the cardiac EPO response. Here, we provide evidence of the hypoxia mimetic, CoCl_2_, stabilized HIF-2α in the heart (Figure 6), which induced downstream local *Epo* production (data not shown). Yasuda et al., report 17β-estradiol- (E_2_) induced *Epo* production in the uterus is not regulated by hypoxia (Yasuda et al., 1998). In another study, potent VEGF inhibition increases hepatic *Epo* synthesis and modulates erythropoiesis in a HIF1α-independent manner (Tam et al., 2006). Our data show cardiac *Epo* overexpression in the EPO^Δ/Δ^ heart was independent of HIF-2α stabilization (Figure 6) and investigations on HIF-1α target genes further suggest that *Epo* regulation was not related to a hypoxic stimulus (Figure 7). To our knowledge, this is the first report of cardiac *Epo* overexpression that occurs independent of HIF-1α and HIF-2α stabilization under normoxic conditions. The identity of the transcription factor(s)/repressors responsible for initiating this phenomenon remain to be solved. Using siRNA knock-down, chromatin immunoprecipitation assays that reveal DNA-protein interactions at the *Epo* enhancer binding sites, or transgenic knockout models, the mechanisms regulating hypoxia-independent cardiac *Epo* production may become apparent.

This study had limitations. We observed no gross organ hypertrophy, though cellular cross-sectional area and posterior wall thickness were increased in alignment with improvements in cardiac function. Considering cross-sectional area of a muscle is proportional to its force output, this seems reasonable. To maintain the same organ weight and size, a cardiomyocyte with increased cross-sectional area would either need to 1) shorten lengthwise or 2) undergo apoptosis. To address the former, in our previous study, we confirmed cardiogenesis is modulated by the presence of cardiac *Epo*–therefore, how long, and wide a cardiomyocyte grows is dependent upon endothelial cell (Colliva et al., 2020) and EPO signaling (Colliva et al., 2020; Allwood et al., 2024). Second, apoptosis is a hallmark feature of the transition from compensatory hypertrophy to heart failure (for review (Van Empel et al., 2005)). Accordingly, reduced contractile function of the heart and increased caspase 8 expression (Kruidering and Evan, 2000) would accompany cardiomyocyte apoptosis, which was not the case for the EPO^Δ/Δ^ mice as confirmed by no difference in the amount of apoptotic cell death by TUNEL assay (Supplementary Figure S5A). Next, while we did not reconcile the transcription factor(s) responsible for regulating hypoxia-independent *Epo* overexpression, we established it was not via the canonical axis involving HIF-1 or HIF-2. Using next-generation chromatin immunoprecipitation assays/sequencing, the protein-DNA interactions that mediate this response might be uncovered. Further, by modifying the *Epo* gene construct, *LacZ-tagged Epo* reporter mice could reveal novel enhancer/promoter regions responsible for cardiac *Epo* expression. (i.e., 5′ kidney inducibility element, 3’ liver inducibility element).

## 5 Conclusion

We have uncovered a novel paradigm wherein adult cardiomyocyte *Epo* deletion induced endothelial cell derived *Epo* and subsequent *Vegfb* expression, which together appeared to stimulate cardiomyocyte hypertrophy in a feedforward manner. Along with more efficient cardiac force generation, EPO^Δ/Δ^ mice demonstrated superior resistance to ischemic-reperfusion injury. Accordingly, there was a complex cellular interplay involving the EPO-EPOR and VEGF-VEGFR transduction pathways, which ultimately modulated cardiac structure and function, though future work is required to fully elucidate the mechanisms involved. Together in the heart, these pathways act in concert.

## Figures and Tables

**Table:** 

**ANP**	atrial natriuretic peptide
**BNP**	brain natriuretic peptide
**Cas8**	caspase 8
**cDNA**	complimentary DNA
**CLAMS**	Comprehensive Laboratory Animal Monitoring System
**CoCl2**	cobalt chloride
**DAPI**	6-diamidino-2-phenylindole
**dP/dtmax**	maximal rate of change of systolic pressure during systole
**dP/dtmin**	maximal rate of change of systolic pressure during diastole
**dP/dt@LVP40**	maximal rate of change of systolic pressure at 40 mmHg
**EDD**	end diastolic dimension
**EDP**	end diastolic pressure
**Eef1e1**	eukaryotic translation elongation factor 1 epsilon 1
**ELISA**	enzyme linked immunosorbent assay
**EPO**	erythropoietin
**EPOR2**	erythropoietin receptor
**ESD**	end systolic dimension
**GLUT**	glucose transporter
**HIF**	hypoxia inducible factor
**HW/BW**	heart weight to body weight ratio
**HW/TL**	heart weight to tibial length ratio
**IP**	intraperitoneal
**kDa**	kilodalton
**kb**	kilobase
**Kdr**	kinase insert domain receptor
**LV**	left ventricle
**LVP**	left ventricular systolic pressure
**αMHC**	alpha myosin heavy chain
**ßMHC**	beta myosin heavy chain
**Myh6**	myosin heavy chain 6
**PGK-1**	phosphoglycerate kinase 1
**qPCR**	real-time quantitative polymerase chain reaction
**RER**	respiratory exchange ratio
**rhEPO**	recombinant human erythropoietin
**Rpl32**	ribosomal protein L32
**SD**	standard deviation
**SDS-PAGE**	sodium dodecyl sulfate-polyacrylamide gel electrophoresis
**SEM**	standard error of the mean
**TMA**	tissue micro array
**VCO2**	volume of carbon dioxide expired
**VO2**	volume of oxygen inspired
**VEGF**	vascular endothelial growth factor
**VEGFR**	vascular endothelial growth factor receptor

## Data Availability

The raw data supporting the conclusion of this article will be made available by the authors, without undue reservation.
