# Supplementary material for: Cardiomyocyte crosstalk with endothelium modulates cardiac structure, function, and ischemia-reperfusion injury susceptibility through erythropoietin
Source: Front Physiol. 2024 Jul 1;15:1397049. doi: 10.3389/fphys.2024.1397049 (PMC11246973; doi:10.3389/fphys.2024.1397049)
Supplement: Supplementary file 1 [file DataSheet1.PDF]

Supplementary Material

1 Supplementary Figures and Tables

1.1 Supplementary Figures

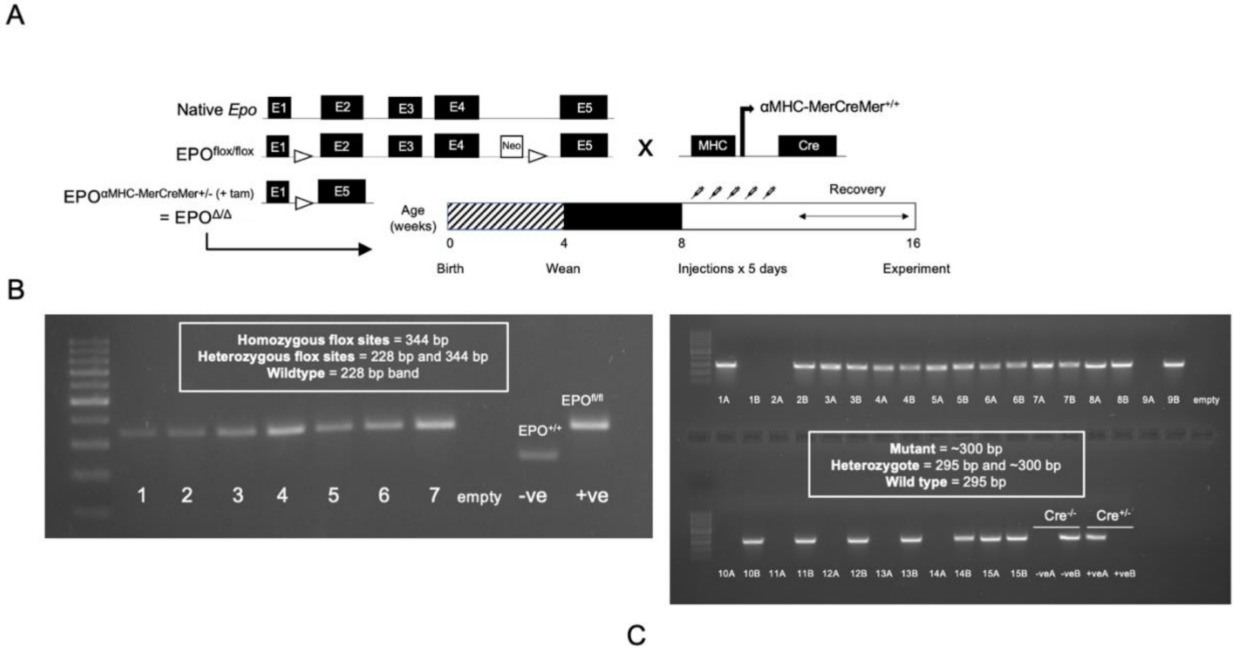

Supplementary Figure 1. Generation and confirmation of the EPO$^{\Delta/\Delta}$ knockout mice by the αMHC-MerCreMer promoter and Cre recombinase expression. A) Schematic of the native *Epo*, EPO$^{fl/fl}$, and EPO$^{\Delta/\Delta}$ alleles. The 5' loxP site was inserted into intron 1 of the mouse *Epo* gene (located 94 base pairs upstream from exon 2). The 3' loxP site was inserted into intron 4 (located 86 base pairs downstream of the exon 4). The NEO cassette, which was flanked by the 5' and 3' loxP sites, was inserted upstream of the 3' loxP site. Experimental mice were generated by crossing homozygous EPO$^{fl/fl}$ female mice with homozygous EPO$^{fl/fl}$: αMHC-MerCreMer$^{+/+}$ male mice to produce mice homozygous for the flox sites and heterozygous for Cre expression (i.e., EPO$^{\alpha MHC\text{-}MerCreMer+/-}$). Once EPO$^{\alpha MHC\text{-}MerCreMer+/-}$ mice were provided tamoxifen (25mg/kg i.p. for 5 days), target mice were generated (i.e., "EPO$^{\Delta/\Delta}$"). No differences were observed in mice not expressing Cre and/or who did not receive tamoxifen injections. Accordingly, these mice were combined and treated as the control group (i.e., "EPO$^{fl/fl}$"). Representative agarose gel images of B) EPO$^{fl/fl}$ and C) αMHC-MerCreMer PCR genotyping.

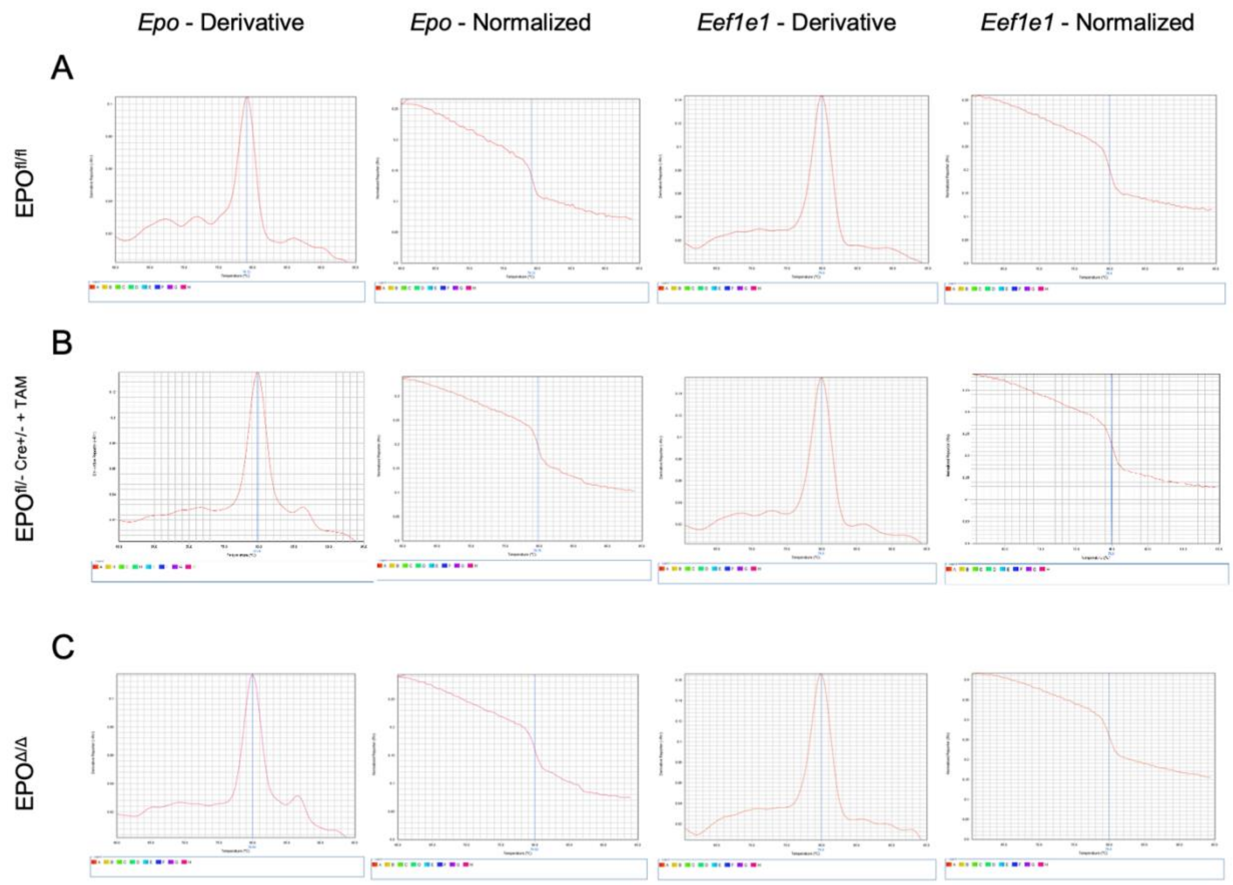

Supplementary Figure 2. *Epo* and HKG primer melting curves. Representative images of the melting curves for *Epo* in the a) $EPO^{fl/fl}$, b) $EPO^{fl/-\ Cre+/-\ +\ TAM}$, and c) $EPO^{\Delta/\Delta}$ mice presented in the form of both the derivative and the normalized reporter.

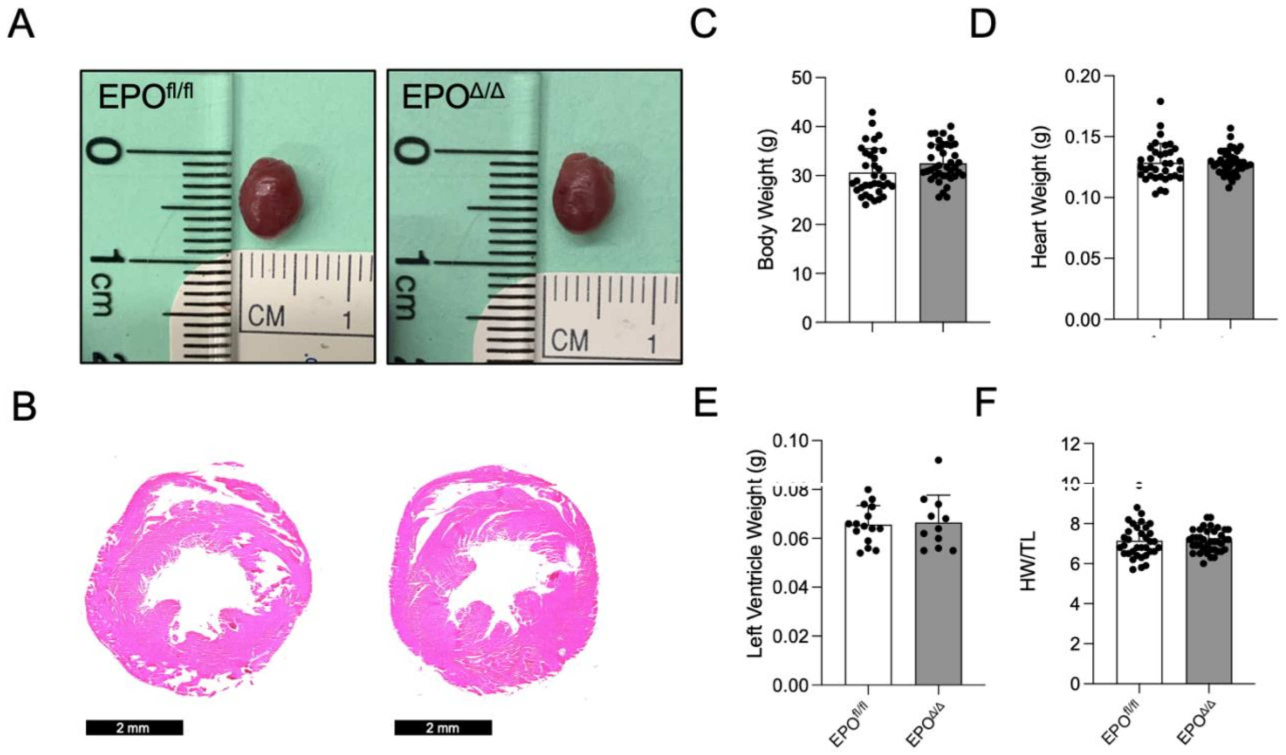

Supplementary Figure 3. Morphological assessments in EPO$^{fl/fl}$ and EPO$^{\Delta/\Delta}$ mice showed no changes in body weight or whole-organ heart weight (raw and normalized to tibial length). A) Representative photos and B) hematoxylin and eosin-stained hearts EPO$^{fl/fl}$ and EPO$^{\Delta/\Delta}$ male mice. Scale bar represents 2mm. C) Body weight, D) heart weight, E) left ventricular weight, F) heart weight/tibial length ratios. An unpaired two-tailed t-test was used to detect differences between EPO$^{fl/fl}$ and EPO$^{\Delta/\Delta}$ mice. Data are expressed as mean ± SD (excluding Figure 2G, which is mean ± SEM). Data were considered significant when P<0.05. HW/TL, heart heart/tibial length ratio.

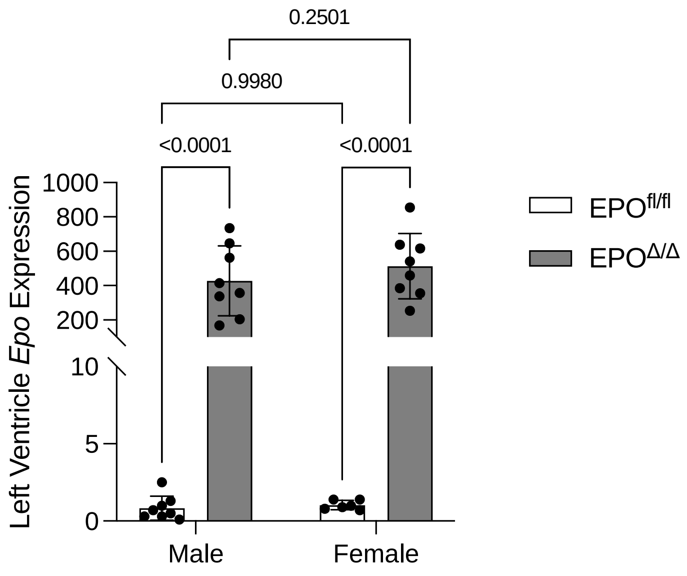

Supplementary Figure 4. ***Epo*** **RNA expression was significantly upregulated in male and female EPO$^{\Delta/\Delta}$ mice, with no apparent sex effect.** Šidák multiple comparisons two-way ANOVA were used

to assess the effect of genotype and sex. Data are expressed as mean ± SD and were considered significant when P<0.05.

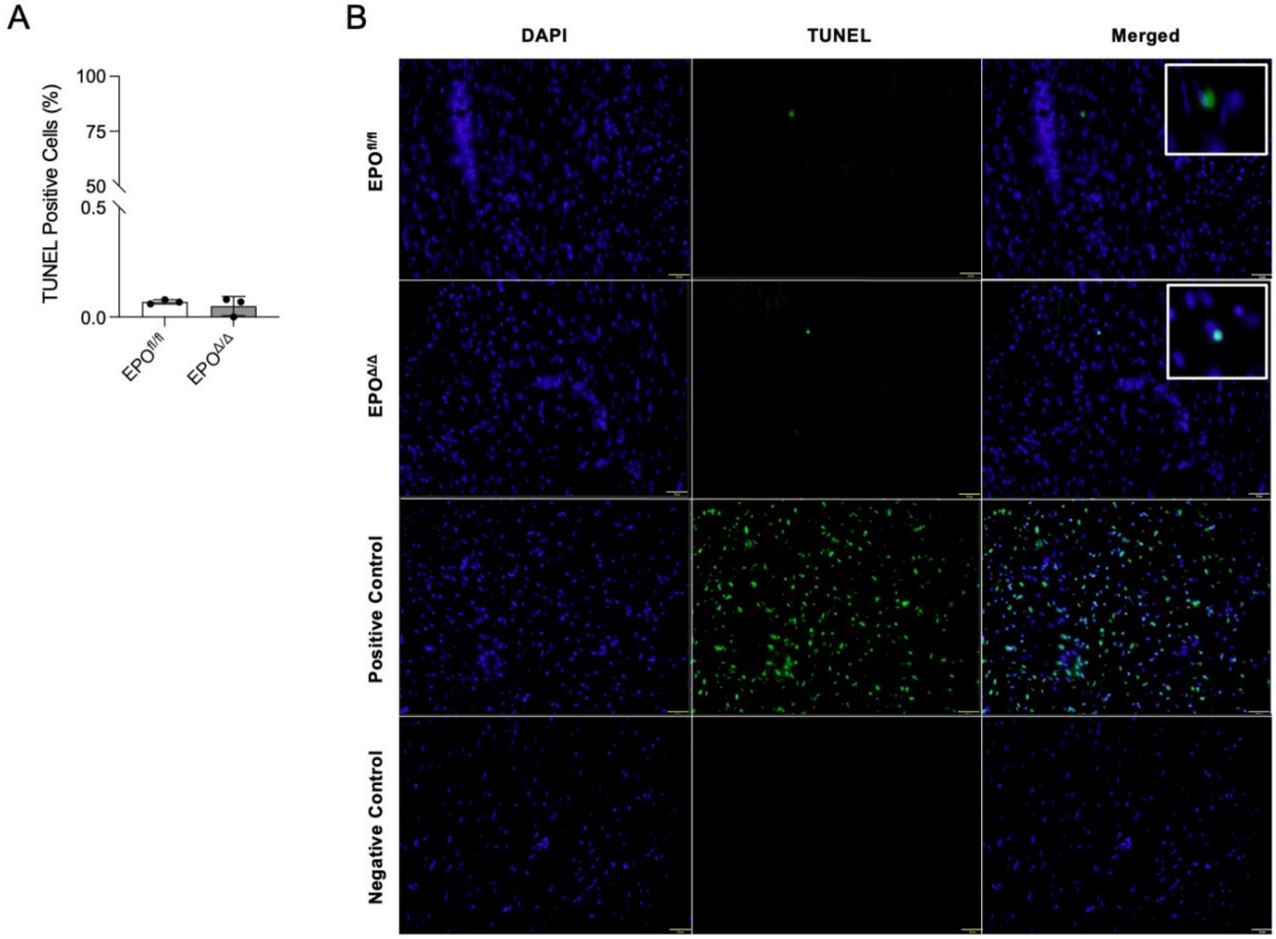

Supplemental Figure 5. TUNEL staining for the assessment of cell apoptosis in the heart. A) Percentage of TUNEL-positive in EPO$^{fl/fl}$ and EPO$^{\Delta/\Delta}$ hearts. B) Representative images acquired at 20X magnification. Scale bar represents 32μm.

1.2 Supplementary Tables

	$Epo^{fl/fl}$ (n=35)	$Epo^{fl/fl + TAM}$ (n=12)	$Epo^{+/+}Cre^{+/- \text{ with or without TAM}}$ (n=20)	$Epo^{fl/+} Cre^{+/- TAM}$ (n=6)	P Value
Body Weight (g)	31±5	32±4	28±3	32±3	0.36, 0.53, 0.80
Heart Weight (mg)	129±16	125±10	126±11	123±5	>0.99, >0.99, >0.99
Tibial Length (cm)	1.8±0.0	1.8±0.0	**1.8±0.0***	1.8±0.0	0.33, **0.05**, 0.65
HW/BW Ratio	4.3±0.4	**3.9±0.3***	4.5±0.5	3.8±0.3	**0.02**, >0.99, 0.06
HW/TL Ratio	7.2±1.0	6.9±0.5	6.9±0.5	6.8±0.2	>0.99, 0.99, 0.93
Spleen (mg)	63±8	N/A	64±12	N/A	N/A, 0.76, N/A
Right Kidney (mg)	158±22	N/A	157±17	N/A	N/A, 0.83, N/A
Liver (g)	1.1±0.1	N/A	**1.3±0.2***	N/A	N/A, **<0.001**, N/A
LV Hematocrit (%)	37±3	N/A	**44±5***	43±2	N/A, **<0.0001**, 0.06

Supplementary Table 1. Table of morphometrics from control groups. A) Morphometrics from various control mice. No differences were measured between $EPO^{+/+}Cre^{+/-}$ mice with or without tamoxifen and thus they were subsequently combined as one group. Mice with one or no floxed sites display higher hematocrits compared to floxed counterparts due to hypomorphic EPO alleles, as previously published[1]. Therefore, these mice were not combined to form the wildtype group (i.e., $EPO^{fl/fl}$). Data are expressed as mean ± SD (morphometrics). P values were compared to $EPO^{fl/fl}$ group as states as "($EPO^{fl/fl + TAM}$, $EPO^{+/+}$ $Cre^{+/- \text{ with and without TAM}}$, $EPO^{fl/+}$ $Cre^{+/- TAM}$)". Data were considered significant when P<0.05.

Left Ventricle (LV)	$EPO^{fl/fl}$ (n=14)	$EPO^{fl/fl + AXI}$ (n=8)	P-value
Heart Rate (bpm)	538±7	**509±9***	**0.02**
LVP (mmHg)	103±2	104±1	0.47
LV EDP (mmHg)	6±1	6±0	0.84
Systolic Pressure (mmHg)	98±2	98±1	0.75
Diastolic Pressure (mmHg)	63±2	58±1	0.10
dP/dt max (mmHg/s)	9840±155	9641±276	0.51
dP/dt min (mmHg/s)	-9337±206	**-8033±281***	**0.002**
dP/dt max @ LVP 40 (mmHg/s)	9297±152	9182±237	0.67
Tau Logistic (ms)	4.3±0.2	N/A	N/A

Supplementary Table 2. Invasive hemodynamic parameters from $EPO^{fl/fl}$ (recalled from Table 4.3) and $EPO^{fl/fl + AXI}$ mice. Data are expressed as mean ± SEM. When P<0.05 (as determined by un-

paired, two-tailed t-test), data were considered significant. Significance was considered when $P<0.05$ compared to $EPO^{fl/fl}$. LVP, left ventricle peak pressure; LV EDP, left ventricle end diastolic pressure.

References

1. Zeigler, B. M., Vajdos, J., Qin, W., Loverro, L. & Niss, K. A mouse model for an erythropoietin-deficiency anemia. *Dis Model Mech* **3**, 763–772 (2010).
